# Baseline cognitive status and adherence are associated with cognitive change following language learning in older adults: an exploratory study

**DOI:** 10.3389/fragi.2026.1734620

**Published:** 2026-08-17

**Authors:** Ladan Ghazi Saidi, Zoha Deldar, Ricardo Carrillo-Meza, Kiley Allgood, Alison Gansemer, Mariah Gentz, Amor Sofia Calderón-Hernández, Yunju Im, Cary R. Savage, Douglas H. Schultz

**Affiliations:** 1 Department of Communication Disorders, College of Education, University of Nebraska at Kearney, Kearney, NE, United States; 2 Center for Brain, Biology, and Behavior, University of Nebraska–Lincoln, Lincoln, NE, United States; 3 Department of Psychology, McGill University, Montreal, QC, Canada; 4 Department of Anatomy, Université Du Québec à Trois-Rivières, Trois-Rivières, QC, Canada; 5 Center of Health Sciences, Autonomous University of Baja California, Mexicali, Mexico; 6 University Center of Health Sciences, University of Guadalajara, Guadalajara, Mexico; 7 Department of Biostatistics, UNMC College of Public Health, University of Nebraska Medical Center, Omaha, NE, United States; 8 Department of Psychology, University of Nebraska–Lincoln, Lincoln, NE, United States

**Keywords:** aging, cognition, cognitive enhancement, cognitive health, intervention, language learning, older adults

## Abstract

**Introduction:**

Given cognitive benefits observed in lifelong bilinguals, a key question is whether L2 learning can boost cognition in later life.

**Methods:**

In a pre–post intervention study, we assessed cognitive effects of learning a new language in older adults as measured by global cognition and domain-specific cognitive tests.

**Results and Discussion:**

While the MMSE, MoCA, and domain-specific measures (Stroop, Simon, nonword repetition, phonemic fluency, and semantic fluency) did not show significant whole-group pre-post change, a number of individual participants improved on all or some of these measures. Further, both linear regression and machine learning models indicated that participants with lower baseline scores on the MMSE, MoCA, phonemic fluency, and semantic fluency showed greater improvement in post-intervention measurements, reflecting greater cognitive gains, with phonemic fluency emerging as the best predictor of intervention outcome. A *post hoc*, adherence-sensitive analysis further indicated a +1.225-point MoCA advantage in completers relative to non-completers, consistent with an adherence-associated pattern. Machine learning analyses confirmed that cognitive gains were heterogeneous across participants and that the most responsive outcomes were reaction-time-based executive measures, particularly the Stroop task and Symbol Digit Substitution Test (SDST). Cluster analysis showed that the top improver profile combined lower baseline executive performance with markedly higher engagement and lesson completion.

**Conclusion:**

These results provide preliminary evidence that engagement in a structured and sustained L2-learning program may be associated with measurable pre–post cognitive changes in some older adults, especially when baseline performance leaves room for improvement. Further, high intervention adherence and engagement are critical to the extent of cognitive benefits derived from L2 learning interventions. Clinically, structured language learning programs that emphasize adherence, dose, and fidelity appear promising for older adults, especially those with emerging decline. These results are noteworthy, given the scarcity of effective options to improve cognitive health in aging, and the fact that L2 learning is low-cost and broadly accessible.

## 1 Introduction

In recent decades, evidence has increasingly supported that lifelong bilingualism is associated with a cognitive advantage relative to monolingualism (Bialystok et al., 2007; Prior and MacWhinney, 2010; Olsen et al., 2015; Bialystok, 2021). Cognitive advantage in proficient bilingual speakers include exhibiting enhanced cognitive resilience against age-related decline (Luk et al., 2011; Gold et al., 2013). This resilience is predominantly observed as enhanced executive functions, including attentional control, problem-solving, and mental flexibility (Stocco and Prat, 2014; Grote et al., 2021). Nevertheless, in a recent systematic review, the authors argued that there was no evidence found for a broad, domain-general bilingual advantage in executive control. However, when executive-function subdomains were analyzed separately, bilingualism was consistently associated with superior inhibitory control, particularly on the Stroop and Simon tasks (Degirmenci et al., 2022). In contrast, other reviews concluded that the cognitive advantage in bilingual individuals is stronger for global cognition and inhibitory control than for other domains, and sensitive to second language (L2) proficiency, acquisition history, and immigration context (Bialystok and Craik, 2022; Chen et al., 2022).

Bilingual advantage also seems to be a protective mechanism against cognitive decline (Bialystok et al., 2007; Bialystok, 2021). In fact, evidence suggests that bilingual individuals, compared to their monolingual peers, demonstrate more robust maintenance of executive functions as they age (Valian, 2015; Woumans et al., 2015). Moreover, a recent meta-analysis reported that the advantage was more pronounced in older adults with mild cognitive impairment than in cognitively healthy peers (Chen et al., 2022). Although once dementia is established, cognitive decline can progress more rapidly, bilingual older adults often perform better than their brain structure would predict and exhibit later clinical onset than monolinguals (Craik et al., 2010; Paulavicius et al., 2020; Bialystok, 2021). In addition, several longitudinal studies have tested this hypothesis, observing delayed onset of dementia symptoms in bilingual individuals compared to monolingual counterparts (Schweizer et al., 2012; Woumans et al., 2015).

Although the mechanisms underlying bilingual cognitive advantage are not fully explained, converging evidence suggests that using L2 may function as ongoing cognitive training leading to enhanced cognitive performance (Wong et al., 2019; Klimova et al., 2020). This sustained mental engagement is proposed to build cognitive reserve and delay typical manifestations of cognitive aging (Gold et al., 2013; Li et al., 2014; Stern et al., 2020; Anderson et al., 2021; Bialystok, 2021). In line with this interpretation, studies using diverse tasks and measurement modalities show that L2 learning induces neuroplastic changes and enhances cognitive performance (Wong et al., 2019; Blanco-Elorrieta and Caramazza, 2021; Nilsson et al., 2021; Meltzer et al., 2023).

Given the cognitive advantages observed in lifelong bilinguals, an important question is whether late-life L2 learning holds promise as a tool for cognitive enhancement. Cognitive enhancement is often attributed to mental stimulation and neural plasticity (Hannan, 2014). It has been argued that the cognitive advantage due to bilingualism is partly induced by the complexities of acquiring a new language, which in turn may bolster cognitive reserve (Antoniou et al., 2013; Gold, 2015; Dash et al., 2017; Dash et al., 2021). Therefore, we hypothesized that L2 learning might have potential as a cognitive intervention for older adults. However, its efficacy and scope are topics that warrant further investigation, considering the complexities and individual variations inherent in both language acquisition and cognitive aging processes.

A growing body of behavioral intervention studies have examined whether L2 learning in older adults can elicit measurable cognitive benefits, focusing primarily on domains such as attention, executive function, and global cognition. Early evidence showed that a one-week intensive Gaelic course improved attentional switching in older adults (Bak et al., 2016), whereas a 35-week program yielded no significant changes in switching ability, underscoring variability in outcomes (Ramos et al., 2017). A 4-week technology-based pilot demonstrated stable MoCA performance during English learning in older adults (Ware et al., 2017), while another 4-week intensive program improved executive functions and wellbeing (Pfenninger and Polz, 2018). Subsequently, a 6-month program produced global cognitive gains relative to active controls (Wong et al., 2019), and large randomized trials have expanded the evidence base, including studies reporting mixed cognitive outcomes across domains (Berggren et al., 2020; Klimova et al., 2020). More recent studies have leveraged 12-week smartphone-based training, showing executive function benefits comparable to Brain-H-Q, a brain training application (Meltzer et al., 2023). Others examined the effect of different pedagogical approaches of L2 learning (i.e., explicit vs. implicit) over 12 weeks on cognitive functioning in older adults. They observed different cognitive outcomes in the two groups for verbal fluency and digit span but not for other tasks (Van Der Ploeg et al., 2023). Others attempted to identify factors affecting executive attention and functions, such as cognitive reserve, baseline L2 proficiency, and baseline global cognition (Grossmann et al., 2023). Baseline cognitive status emerged as an important factor in improving cognition after 3 weeks of L2 learning.

Finally, two randomized trials compared the effect of L2 learning to other interventions. In the first, the authors compared L2 learning to L2 learning combined with aerobic exercise (Qian et al., 2024). Interestingly, they reported that only the L2 learning group showed cognitive improvement as measured by MoCA and IQ (KBIT) scores. The most recent 12-week semi-blind randomized trial in older adults further reported improvements in both cognitive functioning (i.e., episodic memory and flexibility) and psychosocial wellbeing following L2 learning and music training but not following listening to lecture series (Brouwer et al., 2025).

Building on prior evidence of cognitive benefits from L2 learning, we find language acquisition as a cognitively demanding activity that recruits executive control, working memory, attention, and episodic–semantic systems (Ghazi Saidi et al., 2013; Ansaldo et al., 2015; Ghazi-Saidi and Ansaldo, 2015; Ghazi-Saidi and Ansaldo, 2017a; Ghazi-Saidi and Ansaldo, 2017b; Ghazi-Saidi et al., 2015; Ghazi-Saidi et al., 2017; Berroir et al., 2017). This provides a plausible pathway for transfer to domain-general cognition at aging. As discussed above, observational and intervention studies in older adults suggest small-to-moderate gains in executive function, processing speed, and verbal fluency following structured L2 training, with neuroplastic correlates reported in frontoparietal and temporal networks (Schultz et al., 2024). L2 learning also aligns with cognitive-reserve theory (Stern, 2009; Stern, 2021), potentially enhancing resilience to age-related decline by increasing the efficiency and flexibility of control networks. However, prior work is limited by heterogeneous training doses, variable instructional quality, and inconsistent outcome batteries. This leaves open questions about minimally effective duration, targeted mechanisms, and generalizability to everyday functioning. The present study addresses these gaps by implementing an online L2 program accessible at home in healthy older adults and assessing both domain-specific (executive function components) and global cognition, with the expectation that L2 training will yield measurable improvements consistent with cognitive transfer effects.

In the present study, we investigated the cognitive effects of L2 learning in healthy older adults after 4 months of an L2 learning intervention. We measured both domain-specific and global cognition before and after the language learning intervention. We expected improvements in executive function performance (e.g., conflict management, inhibition, working memory) and in global cognition, reflecting transfer from language learning demands to broader cognitive control and overall cognitive status. Contingent on observing these gains, we hypothesized that L2 learning could serve as a practical, scalable cognitive intervention for older adults, particularly relevant to dementia care and prevention approaches.

## 2 Methods

This study was an exploratory investigation; an approach commonly adopted in emerging intervention domains prior to the implementation of large-scale randomized controlled trials. The present study aimed to evaluate the feasibility of implementing a second language learning intervention in cognitively healthy older monolingual adults and to examine whether measurable pre-post changes in cognitive performance could be detected over a relatively short intervention period. In addition to assessing potential cognitive changes, the study was designed to provide preliminary evidence regarding participant adherence, feasibility of the intervention protocol, and the magnitude and variability of potential cognitive effects. Such early-stage investigations are essential for establishing the methodological foundation and informing the design, implementation, and statistical power considerations of future rigorously controlled trials examining language learning as a potential cognitive intervention in aging.

### 2.1 Design

We explored the cognitive effects of L2 learning in older adults residing in Nebraska using a pre-post intervention design. The intervention was administered for 4 months, with sessions of 60–90 min per day, at least 5 days a week. We assessed global cognition using two widely used clinical screening tools for aging-related cognitive decline: the Montreal Cognitive Assessment (MoCA) and Mini-Mental State Examination (MMSE). Additionally, we explored specific cognitive domains: executive functions, attention, verbal and nonverbal memory, using some of the most commonly used tasks in cognitive research: the Stroop task, the Simon task, the Symbol Digit Substitution Test (SDST), as well as nonword-repetition and semantic and phonological verbal fluency.

### 2.2 Participants

The initial recruitment pool consisted of 286 individuals who showed interest in the study, 201 of whom responded to our screening survey. A total of 37 volunteers were excluded because they did not meet the inclusion criteria, 75 volunteers never responded back and 38 participants decided to drop out of the study due to personal choice, or finding the intervention too time consuming. Thus, the final analytic sample included 45 participants. This reduction to the final sample resulted from the application of strict inclusion criteria, including cognitively healthy status, monolingual background, adherence to the intervention protocol, and availability of complete datasets. These criteria were applied to maintain methodological rigor and minimize potential confounding factors in this exploratory investigation. The 45 participants who completed the study comprised healthy monolingual adults, aged between 60 and 80 years, of all genders, residing in a predominantly monolingual environment in Nebraska. Restricting the sample to monolingual participants minimized the potential influence of prior bilingual experience, which has been associated with cognitive reserve and cognitive advantages in aging. This approach reduces a potential confounding factor and allows for a clearer examination of whether initiating second language learning later in life is associated with cognitive changes. The inclusion criteria included being a monolingual English-speaking adult aged 60–80 years of any gender, race, or ethnicity, having access to the internet and an electronic device, and possessing normal or corrected vision and hearing, and motivation to commit to our language learning intervention for the duration of the study. Exclusion criteria included memory problems or learning disabilities, a diagnosis of depression, or any neurological disorders. Participants with a MoCA score below 23 were excluded. See Table 1 for demographic characteristics of the participants as well as their MoCA scores at pre- and post-intervention.

**TABLE 1:** Participant demographics and cognitive scores at baseline and post-intervention.

Characteristic	Minimum	Maximum	M	SD
Age (years)	60	77	66.58	4.55
MoCA				
Pre-intervention	24	30	27.73	1.70
Post-intervention	23	30	27.82	1.62
MMSE				
Pre-intervention	28	30	29.67	0.60
Post-intervention	25	30	29.11	1.15
Education				
Bachelor’s	20 (44.44%)	​	​	​
Master’s	13 (28.89%)	​	​	​
Doctorate	7 (15.56%)	​	​	​
Some college	5 (11.11%)	​	​	​
Gender				
Female	32 (71.11%)	​	​	​
Male	13 (28.89%)	​	​	​

*N* = total number of participants; *M* = mean; *SD*, standard deviation; MoCA, montreal cognitive assessment; MMSE, Mini-Mental State Examination. for education and gender, values are reported as *n* (frequency) and % (percentage). Values are based on the full sample (*N* = 45).

#### 2.2.1 Second language proficiency screening

All participants were screened for their knowledge, exposure, and use of other languages using the Language Experience and Proficiency Questionnaire (LEAP-Q) (Marian et al., 2007). LEAP-Q is a self-report toolbox that collects data on language knowledge, use, and exposure for all languages spoken by an individual through family members, friends, education, travel, radio, television, music and cultural contexts (Marian et al., 2007; Kaushanskaya et al., 2020). Scores range 0–10, and scores ≥7 are taken as bilingual proficiency; a score of 0 indicates monolingualism. Only participants with a score of 0 or 1 were included. All participants were U.S.-born American adults who were born and raised in monolingual English-speaking households and resided in Nebraska, a predominantly monolingual environment with limited routine exposure to languages other than English. LEAP-Q responses furt her confirmed that participants reported no functional use of another language and minimal prior exposure to the target language or associated cultural contexts. LEAP-Q responses were used to confirm functional monolingual status and minimal prior exposure to the target language and associated cultural context. A score of 1 on the relevant LEAP-Q exposure/use items indicated no meaningful exposure to or use of the language. Participants who reported scores of 2 or higher, indicating any meaningful exposure, use, or proficiency in another language, were excluded from the study. Thus, the final sample included only participants who reported no functional use of another language and minimal exposure to the target language or related cultural contexts.

### 2.3 Procedure

The study protocol was approved by the University of Nebraska–Lincoln Institutional Review Board (IRB; Protocol # UNL-00020756). Participants were recruited via social media posts, newspaper and magazine ads, and word of mouth. Participants were screened via email. Those who met the inclusion criteria signed a consent form and completed Qualtrics questionnaires to provide their demographic information. Subsequently, they were scheduled for a pre-intervention cognitive assessment battery.

Participants were then given access to a Rosetta Stone account for a language they chose from 18 different options. Participants engaged in language learning for 60–90 min a day, 5 days a week, for 4 months. The Rosetta Stone, Education Edition, enabled monitoring of performance metrics and adherence. The online modality provided flexibility, enhancing the study’s generalizability and potential for real-world application.

### 2.4 Pre- and post-cognitive assessment battery

We used cognitive screening tools that are common in both research and the clinic to assess global cognition and executive functions, attention, and verbal and nonverbal memory. We also selected tasks known to be sensitive to bilingual cognitive advantage and/or cognitive decline. This battery evaluated cognitive domains such as executive functions, memory, working memory, and attention, and included tests sensitive to cognitive decline.

By leveraging these diverse yet synergistic assessments, we aimed to provide a robust and nuanced evaluation of cognitive performance across multiple domains. Our assessment approach was rigorous and grounded in two primary rationales: 1) evidence supporting the cognitive benefits of lifelong bilingualism, and 2) the sensitivity of selected tests for early diagnosis of cognitive decline in alignment with prevailing models of aging and cognition. Speed of processing and cognitive capacities such as working memory and inhibitory control are critical for differentiating both age-related and individual variations in cognitive performance. The cognitive assessment battery included several measures. Global cognitive status was assessed using both the Montreal Cognitive Assessment (MoCA) and the Mini-Mental State Examination (MMSE). MoCA and MMSE, two cognitive screening tools sensitive to mild cognitive impairment, served as measures for global cognitive performance and cognitive reserve. The MMSE serves as a practical tool for identifying and tracking cognitive decline, particularly in conditions such as Alzheimer’s disease, vascular dementia, and delirium (Arevalo-Rodriguez et al., 2015). The MoCA (Nasreddine et al., 2005) is a widely used screening tool designed to detect cognitive impairments. It evaluates multiple cognitive domains, including memory, attention, language, visuospatial skills, executive functions, and orientation. The MoCA is particularly sensitive to mild cognitive impairment and early stages of dementia, offering a quick and comprehensive overview of an individual’s cognitive health (Julayanont and Nasreddine, 2017). Unlike the MMSE, which is widely used to diagnose and monitor more advanced cognitive impairment, such as dementia, the MoCA is more effective at identifying subtle deficits. The MMSE (Tombaugh and McIntyre, 1992) is a brief, structured assessment tool used to evaluate cognitive function and screen for cognitive impairment. It measures various cognitive domains, including orientation, registration, attention and calculation, recall, language, and visuospatial skills. The MMSE is commonly used in clinical and research settings to monitor changes in cognitive status over time, particularly in conditions such as dementia (Arevalo-Rodriguez et al., 2015). These instruments provide complementary indices of cognition; the MoCA includes more demanding tasks targeting executive function, attention, and visuospatial processing and is generally considered more sensitive to subtle cognitive changes in high-functioning individuals, whereas the MMSE emphasizes orientation, memory, and basic language functions and provides a widely used indicator of general cognitive status. We administered both the MoCA and MMSE to leverage their complementary strengths; the MoCA’s greater sensitivity to subtle executive changes and MMSE’s comparability and global status, increasing detection power across time, and mitigating ceiling/floor effects.

Domain-specific measures of executive function and working memory were administered via the Gorilla Experiment Builder platform (Anwyl-Irvine et al., 2020). We used the Stroop task (Stroop, 1935), which measures selective attention and executive function (Liu et al., 2004; Gajewski and Falkenstein, 2012; Perrochon et al., 2013; Huang et al., 2020). The Stroop task is a widely recognized tool for assessing executive function and is particularly useful for evaluating selective attention, cognitive flexibility, and response inhibition (MacLeod, 1991). We also used the Simon task (Simon and Rudell, 1967), which assesses interference/conflict management (Simon and Rudell, 1967; Liu et al., 2004; Kerns, 2006). While the Stroop task is language-based, the Simon task is non-verbal. The Simon task is a widely used measure to assess cognitive control, particularly useful in evaluating response inhibition and the ability to resolve stimulus-response conflicts. This task requires participants to respond based on the attributes of the stimulus while ignoring irrelevant spatial information, thereby assessing how effectively one can suppress automatic and conflicting responses (Lu and Proctor, 1995). Finally, we used the SDST (Hinton-Bayre and Geffen, 2005), which assesses processing speed (Joy et al., 2004; Silva et al., 2018). The SDST is a brief, validated neuropsychological measure designed to assess processing speed, attention, visual scanning, and motor speed, making it particularly sensitive for detecting subtle cognitive impairment in conditions such as multiple sclerosis, traumatic brain injury, and aging (Smith, 1982; Benedict et al., 2017).

We also administered nonword repetition, semantic and phonemic fluency tests, which evaluate verbal working memory (Azuma, 2004; Oh et al., 2019), and nonword repetition, which measures verbal working memory (Baddeley, 1992; Baddeley et al., 1998; Gathercole, 2006). Nonword repetition is a widely recognized tool in neuropsychology used to assess phonological working memory and language processing (Gathercole et al., 1994). This task involves listening to and repeating phonological sequences that carry no meaning, making it especially useful for evaluating sublexical phonological skills independently of vocabulary knowledge. It allows for the examination of short-term verbal memory integrity and phonological encoding, both of which are fundamental to language learning and acquisition (Gathercole et al., 1994). Phonemic verbal fluency is a widely used neuropsychological test designed to evaluate executive functions, particularly those associated with frontal lobe activity, as well as language production and processing speed. The task involves asking participants to produce as many words as possible that begin with a specific letter, within a 1-min time limit. This requires the coordination of multiple cognitive processes, including lexical access, working memory, cognitive flexibility, inhibitory control, and self-monitoring. Due to its sensitivity to prefrontal cortex function, this test is commonly used in clinical settings to detect early signs of cognitive decline, such as in Alzheimer’s disease, frontotemporal dementia, traumatic brain injury, or certain psychiatric disorders (Henry and Crawford, 2004). Semantic verbal fluency assesses the efficiency of lexical access and the organization of semantic memory by asking participants to generate as many words as possible within a specific semantic category under time constraints (Henry and Crawford, 2004).

Higher scores on the Mini-Mental State Examination (MMSE) and Montreal Cognitive Assessment (MoCA) indicate better global cognitive functioning. For verbal fluency, higher scores indicate better lexical retrieval and verbal executive functioning, reflected by the production of a greater number of correct words within the allotted time. For the nonword repetition task, higher scores indicate better phonological working memory and speech-sound processing, reflected by a greater number of accurately repeated nonwords. For the Simon task, greater accuracy and/or faster reaction times indicate better inhibitory control and response selection; larger interference effects, reflected by slower or less accurate responses on incongruent relative to congruent trials, indicate greater difficulty with cognitive control. For the Stroop task, greater accuracy and/or faster reaction times indicate better selective attention and inhibitory control; larger Stroop interference effects, reflected by slower or less accurate responses on incongruent relative to congruent trials, indicate greater inhibitory control demands. For the Symbol Digit Substitution Test, higher scores indicate better processing speed and visual-motor coordination, reflected by a greater number of correct symbol digit pairings completed within the time limit.

### 2.5 Intervention

The L2 learning intervention was administered via the educational version of Rosetta Stone. Participants were provided with the instructions, documents, and research assistants facilitated the initial setup and provided ongoing support, if required. Each participant engaged in the online program self-paced and independently. Rosetta Stone online language program employs the Communicative Language Teaching (CLT) method (Thompson, 1996). Rosetta Stone, recognized as a gold standard in adult language learning, offered several advantages, including high user acceptability rates (80%–90%). The CLT method, employed by Rosetta, was considered effective and efficient for L2 acquisition (Thompson, 1996; Vesselinov, 2009; DeWaard, 2013; Lord, 2016). The platform featured real-world scenarios, practice opportunities, and immediate feedback. It includes baseline placement tests and progressive mastery assessments, which were essential for tracking participants’ linguistic progress. The Rosetta program estimated core lessons at 30 min each, focused activities between 5–10 min, and milestones around 15 min. The intervention dose was 60–90 min per day, 5 days a week, over a four-month period, with no restriction on the maximum amount of time they could invest. This approach was based on the understanding that increased exposure and use are crucial for the neurocognitive benefits of language learning, as well as balancing between a minimum period sufficient for consolidated learning and eliciting behavioral change, while remaining brief enough to minimize participant burden and maximize compliance and adherence (Ghazi Saidi et al., 2013; Ansaldo et al., 2015; Ghazi-Saidi and Ansaldo, 2015; Ghazi-Saidi and Ansaldo, 2017a; Ghazi-Saidi and Ansaldo, 2017b; Ghazi-Saidi et al., 2015; Ghazi-Saidi et al., 2017; Berroir et al., 2017; Surrain and Luk, 2019).

The educational version of the language platform allowed for systematic monitoring of participants’ engagement, performance, and overall intervention compliance. For adherence and data integrity, research assistants had administrative access to activity metrics, which were downloadable via Excel and enabled detailed monitoring of participant engagement. Specifically, adherence was tracked through administrative login checks, weekly performance reports, and objective platform-based metrics, including minutes per day, minutes per week, and total cumulative minutes across the intervention period. To support adherence, research assistants sent weekly or biweekly email reminders and conducted monthly group meetings via Zoom, while optional Zoom check-in sessions provided additional opportunities to address questions and obtain participant self-assessments of progress. Non-adherence protocols were also implemented: participants who did not engage with the program for more than 1 week were contacted, and those who remained inactive for more than 2 weeks without response were excluded from the study. In addition to engagement tracking, the platform included embedded assessments at the end of each lesson and level, which were used to document learning progress and proficiency levels, and course completion rates were also recorded. Finally, at the end of the intervention, participants completed an exit survey assessing their perceptions of learning and language use.

Rosetta Stone was used as a standardized, accessible, computer-based tool to deliver the L2-learning intervention. Thus, the cognitive outcomes were selected based on the broader cognitive demands of adult L2 learning, including lexical acquisition, phonological processing, working memory, attention, processing speed, and executive control. The core demands of L2 learning, as implemented through the computer-based training program, map onto the selected outcome measures. Specifically, vocabulary acquisition and lexical retrieval were expected to engage verbal fluency; perception and repetition of unfamiliar sound patterns were expected to engage phonological working memory, measured by nonword repetition; response selection and suppression of competing alternatives were expected to engage inhibitory control, measured by the Simon and Stroop tasks; speeded visual-linguistic mapping and response generation were expected to engage processing speed, measured by the Symbol Digit Substitution Test; and global cognitive screeners were included to characterize broad cognitive status and monitor general pre-post cognitive change.

### 2.6 Data analysis

First, parametric inferential statistics within the general linear model (t-test, ANOVA, and linear regression) were conducted to examine the pre–post effect as well as relationships between pre–post scores and pre–post improvement. Then, to assess patterns of cognitive improvement and identify their predictors, we implemented a comprehensive analytical machine-learning approach. Finally, we conducted *post hoc* analyses to evaluate the machine-learning results.

Engagement with the intervention program was measured through time-based metrics and completion metrics. Time-based metrics included average days logged in per week, average time per week (minutes), cumulative time (minutes), and average time per day (minutes). The completion metrics included completion percentages for program levels (Level 1, Level 2, Level 3), and average completion percentage across levels.

#### 2.6.1 ANOVAs and regression analysis

Within-participant change on each cognitive measure (MoCA, MMSE, Stroop, Simon, SDST, Semantic Fluency, Phonemic Fluency, Nonword Repetition) was evaluated with paired-samples *t* tests comparing pre- (T1) and post-intervention (T2) scores in the full sample (*N* = 45). For Stroop and Simon, repeated-measures ANOVAs tested main effects of session (T1 vs. T2), condition (congruent, incongruent, neutral), and their interaction. Family-wise error was controlled using the Holm–Bonferroni procedure applied within each analytic family, and adjusted *p* values are reported. Linear regressions examined associations between baseline performance and (a) post-intervention scores and (b) improvement (T2 − T1). All analyses were conducted in Python (pandas, statsmodels, Pingouin).

#### 2.6.2 Post hoc analyses

We performed exploratory pre–post comparisons of MoCA and MMSE among participants who completed all three training levels and fit linear models to evaluate predictors of change (completion status, age, gender). The *p* values for these exploratory comparisons were Bonferroni-corrected.

#### 2.6.3 Machine-learning analysis

Given the variability in cognitive outcomes, we conducted machine-learning analyses to identify the predictors of cognitive change, aiming to determine which factors were most strongly associated with improvement following the intervention. Our machine-learning analyses were designed to address three core questions: (1) which participant characteristics and program engagement metrics predict cognitive improvement, (2) whether distinct patterns of improvement can be identified across participants. To answer these questions, we implemented a comprehensive analytical approach to assess cognitive improvement and identify its predictors, and (3) whether baseline cognitive performance influences the magnitude of improvement.

All data were preprocessed by converting numeric fields to appropriate formats and standardizing column names to remove trailing spaces. For each cognitive measure, improvement was computed as the pre–post difference with directionality aligned to construct meaning: for reaction time (RT) measures, improvement = Pre − Post (lower RT indicates better performance); for accuracy (AR) measures and other cognitive tests (MMSE, MoCA, fluency), improvement = Post − Pre (higher scores indicate better performance). To accommodate disparate scales, all improvement scores were standardized using z-score transformation (*M* = 0, *SD* = 1), and a composite improvement score was derived by averaging standardized improvements across measures to yield a global index of cognitive change. Participants were classified as showing “significant improvement” if their composite score exceeded 0.2 standard deviations; additionally, the percentage of measures showing improvement was computed per participant to enable tertile-based grouping into low, moderate, and high improvement categories. We employed a composite improvement score because it reduces multiple testing (single outcome vs. 23 measures), increases statistical power by pooling information across measures, captures general, rather than task-specific, cognitive change, and aligns with standard practice in major cognitive training studies. Creating a composite score is a well-established and accepted approach in cognitive intervention research (Jaeggi et al., 2008; Shipstead et al., 2012; Melby-Lervåg and Hulme, 2013; Karbach and Verhaeghen, 2014; Moreau and Wiebels, 2021; van Dierendonck and Lam, 2023).

Pearson’s correlation coefficients were calculated between all potential predictors (demographic variables and engagement metrics) and the composite improvement score. Statistical significance was assessed at *p* < 0.05, with trends noted at *p* < 0.10.

An unsupervised machine-learning approach (k-means) employed to identify naturally occurring patterns of improvement within the data, an explicitly exploratory goal. K-means clustering is an unsupervised machine-learning algorithm used to identify patterns or natural groupings within a dataset based on similarities among data points. This group’s participants are divided into k clusters by repeatedly assigning each person to the closest center (centroid) and recalculating those centers until the clusters are as tight as possible. The optimal number of clusters was determined using silhouette score analysis, testing solutions from 2 to 5 clusters. Silhouette is a score that shows how well each person fits in their current cluster compared with the next-closest cluster. If the average score is higher, members are much closer to their own cluster center than to any other cluster, meaning clusters are tighter inside and farther apart from each other. After identifying clusters, their characteristics were compared on improvement patterns and predictor variables. For reproducibility, we specified how improvement scores were calculated for each measure, how the composite improvement index was derived, how participants were grouped on the basis of improvement patterns, how the cluster solution was selected, and how PCA was used to visualize the resulting response structure.

For each cognitive measure, Pearson correlations were calculated between baseline (Pre) performance and improvement magnitude. Additionally, to characterize patterns associated with the greatest gains, baseline performance of the top 25% of improvers was compared to the rest of the sample using descriptive statistics. Independent samples t-tests were used to compare improvement between gender groups. Correlational analysis was used to examine the relationship between age and improvement. This approach is consistent with other major studies in the field (Jaeggi et al., 2008; Shipstead et al., 2012; Melby-Lervåg and Hulme, 2013; Karbach and Verhaeghen, 2014; Moreau and Wiebels, 2021; van Dierendonck and Lam, 2023).

These data-driven analyses were intended to characterize heterogeneity in intervention response within the sample rather than to develop a formal clinical prediction algorithm. Accordingly, the clustering solution was evaluated primarily on the basis of internal separation, as indexed by silhouette scores, together with the interpretability of the resulting response profiles. Principal component analysis (PCA) was then used as a complementary visualization approach to display the separation of participant patterns in reduced-dimensional space.

All analyses were conducted using Python 3. x with scientific computing libraries (NumPy, pandas, SciPy, Pingouin) and machine-learning tools (scikit-learn) for cluster analysis and standardization. Multiple visualization techniques were employed to represent findings, including histograms, scatter plots, correlation heatmaps, and principal component analysis plots for cluster visualization.

## 3 Results

### 3.1 Participant descriptive statistics

A total of 45 participants were enrolled in the study. Participants ranged in age from 60 to 80 years, with a mean age of 66.6 years (*SD* = 4.52). The sample was predominantly female, with approximately 71% identifying as female and 29% as male. See Table 1 for details.

### 3.2 Intervention adherence results

Participants chose Spanish (n = 39), German (2), French (n = 2), Italian (n = 1), and Japanese (n = 1). Participants demonstrated a high level of engagement with the language learning intervention. On average, individuals spent 65.5 min per day (*SD =* 23.8) engaged in language practice. The cumulative time dedicated to learning throughout the intervention period was 4858 min (approximately 81 h), with a standard deviation of 2398 min, indicating moderate variability in overall time investment across participants. In terms of curriculum progression, 92.9% of Level 1, 77.0% of Level 2, and 59.5% of Level 3 were completed on average. When averaging progress across levels, the mean completion rate was 76.5% (*SD =* 28.0%), suggesting a generally strong adherence to the intervention, albeit with some individual variation in the extent of lesson completion.

Adherence analyses indicated sustained engagement with the intervention, as reflected in regular log-ins and documented time spent on the learning platform throughout the intervention period. Embedded lesson and level assessments demonstrated that participants progressed through the program and achieved measurable proficiency milestones. Course completion rates further supported adherence to the intervention protocol. Participant-reported outcomes collected during optional Zoom check-ins and the exit survey indicated that 75% of participants reported learning and using elements of the new language, suggesting meaningful engagement with the program.

### 3.3 Cognitive assessment results

#### 3.3.1 Pre-post comparisons and regression analyses

Cognitive performance across all measures at baseline (T1) and following the intervention (T2) is summarized in Table 1. Pre- (T1) and post-intervention (T2) performances on the MoCA, MMSE, Stroop task, Simon task, SDST, Semantic Fluency, Phonemic Fluency, and Nonword Repetition for all 45 participants are summarized in Table 2.

**TABLE 2:** Pre-intervention (T1) and post-intervention (T2) across cognitive tests.

Cognitive measure	Pre-Intervention *M* (*SD*)	Post-Intervention *M* (*SD*)	*t*	*p*	Cohen’s *d*
MoCA (points)	27.733 (1.70)	27.82 (1.61)	0.621	0.538	0.093
MMSE (points)	29.667 (0.60)	29.11 (1.15)	−3.21	0.002	−0.479
Stroop test (RT, ms)					
Congruent	946.470 (194.824)	947.01 (179.355)	0.017	0.987	0.002
Incongruent	1086.13 (201.35)	1079.76 (165.10)	−0.205	0.838	−0.031
Neutral	942.240 (189.791)	953.08 (179.55)	0.329	0.744	0.049
Average RT	991.615 (190.997)	993.25 (169.39)	0.052	0.959	0.008
Stroop test (AR)					
Congruent	0.930 (0.102)	0.948 (0.080)	0.990	0.328	0.148
Incongruent	0.745 (0.170)	0.786 (0.150)	1.490	0.143	0.222
Neutral	0.921 (0.111)	0.947 (0.093)	1.171	0.248	0.174
Average accuracy	0.866 (0.110)	0.895 (0.099)	1.435	0.158	0.214
Simon task (RT, ms)					
Congruent	948.536 (236.315)	973.495 (239.76)	0.627	0.534	0.093
Incongruent	1081.10 (240.73)	1091.21 (236.92)	0.269	0.789	0.040
Neutral	977.49 (234.61)	1015.98 (235.19)	1.013	0.316	0.151
Average RT	1002.35 (234.19)	1026.81 (234.53)	0.647	0.521	0.096
Simon task (AR)					
Congruent	0.890 (0.15)	0.937 (0.12)	1.560	0.126	0.233
Incongruent	0.820 (0.19)	0.843 (0.14)	0.673	0.504	0.100
Neutral	0.885 (0.15)	0.916 (0.09)	1.222	0.228	0.182
Average accuracy	0.864 (0.16)	0.899 (0.11)	1.236	0.223	0.184
Symbol digit (RT, ms)	1478.05 (280.88)	1483.51 (267.43)	0.128	0.899	0.019
Symbol digit (AR)	0.992 (0.01)	0.993 (0.01)	0.234	0.816	0.035
Semantic fluency (words)	23.489 (5.41)	23.800 (5.31)	0.431	0.669	0.064
Phonemic fluency (words)	14.29 (3.64)	14.38 (3.58)	0.171	0.865	0.026
Nonword repetition (proportion correct)	0.888 (0.09)	0.864 (0.14)	−1.170	0.248	−0.174

*M*, mean; *SD*, standard deviation; RT, reaction time (ms); AR, accuracy (proportion correct). MoCA, Montreal Cognitive Assessment (points); MMSE, Mini-Mental State Examination (points). Semantic and phonemic fluency are reported as the number of words produced. Nonword repetition is reported as proportion correct. Values are based on paired comparisons for the full sample (*N* = 45).

##### 3.3.1.1 Global cognition

###### 3.3.1.1.1 MMSE

A paired-samples t-test comparing MMSE scores before and after the intervention showed a statistically significant pre-post difference, with scores lower at post-intervention (M = 29.11, SD = 1.15) than at pre-intervention (M = 29.67, SD = 0.60), t (44) = 3.22, p = 0.002, d = 0.48. Thus, the raw MMSE means indicate a decline over time.

A simple linear regression was performed to determine whether baseline MMSE scores influenced post-intervention outcomes. This analysis indicated a marginally significant positive association between pre- and post-intervention MMSE scores, F (1, 43) = 2.88, p = 0.097, *R* ^2^ = 0.063, suggesting limited rank-order stability within a restricted score range.

A separate regression analysis was performed using change scores (post minus pre) as the dependent variable, revealing a marginally significant negative relationship with baseline MMSE scores, F (1, 43) = 3.41, p = 0.072, *R* ^2^ = 0.073. This pattern suggests that participants with lower initial MMSE scores tended to show relatively more favorable change over time. Given the narrow baseline range and ceiling constraints of the MMSE in this healthy sample, however, this pattern should be interpreted cautiously.

###### 3.3.1.1.2 MoCA

An initial paired-samples t-test, performed to assess the average treatment effect, revealed no statistically significant difference between pre- and post-intervention MoCA scores, *t* (44) = −0.41, *p* = 0.681. This negligible effect size (*d* = −0.05) indicates that the intervention did not produce a reliable change in global cognitive performance as measured by the MoCA at the group level.

However, recognizing that overall means can mask individual variability and that it is crucial to investigate individual heterogeneity, two subsequent linear regression analyses were conducted. The first regression showed a highly significant positive relationship between pre- and post-intervention scores, *F* (1, 43) = 27.01, *p* < 0.001, *R* ^*2*^ = 0.386. This robust result serves primarily to confirm the psychometric stability of the MoCA, demonstrating strong test-retest reliability and baseline scores’ expected role in predicting post-intervention status.

**FIGURE 1:**
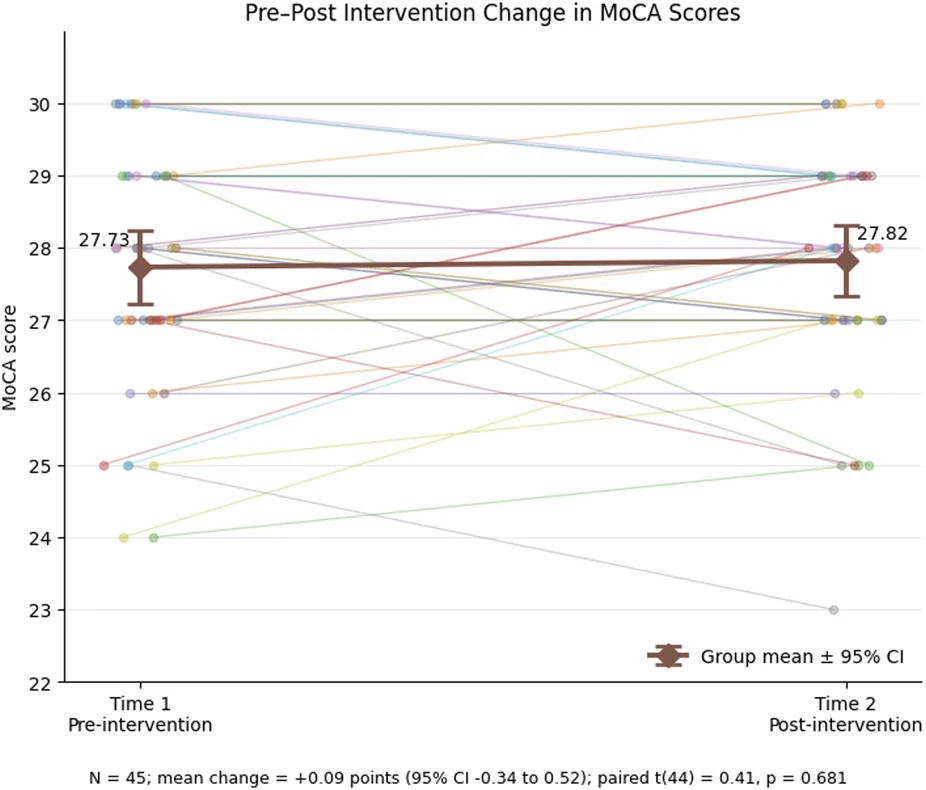
Baseline MoCA scores strongly predict post-intervention performance despite no overall change. Montreal Cognitive Assessment (MoCA) scores before and after the 4-month intervention. Individual trajectories (gray lines) and the mean trend (bold black line) are shown from pre-intervention (T1) to post-intervention (T2). Mean MoCA scores were 27.73 ± 1.70 at T1 and 27.82 ± 1.61 at T2, indicating no significant group-level change in global cognition.

The critical finding, however, was obtained from the analysis of differential gain. A separate regression on the improvement scores (post minus pre) revealed a significant negative association with baseline MoCA scores, *F* (1, 43) = 13.00, *p* < 0.001, *R* ^*2*^ = 0.232. This pattern suggests that despite the non-significant group mean change, participants with lower initial MoCA scores achieved significantly greater absolute cognitive gains over the course of the intervention (see Figure 1). These findings suggest that participants with lower baseline cognitive status exhibited greater improvement over time.

#### 3.3.2 Domain-specific pre–post changes

##### 3.3.2.1 Stroop task (RT, AR), cog/incog/neut

Analysis of Stroop task performance revealed no significant main effect of session (pre-vs-post) on accuracy, *F* (1, 44) = 1.91, *p* = 0.174, *η* ^*2G*^ = 0.018, indicating that overall accuracy did not change significantly from pre-to post-intervention. However, a significant main effect of condition was found, *F* (2, 88) = 109.10, *p* < 0.001, *η* ^*2G*^ = 0.427. *Post hoc* comparisons indicated significantly lower accuracy for incongruent trials relative to both congruent (*p* < 0.001) and neutral trials (*p* < 0.001). There was no significant session × condition interaction, *F* (2, 88) = 0.78, *p* = 0.459, *η* ^*2G*^ = 0.001, suggesting that the intervention did not differentially impact accuracy across trial types.

Similarly, analysis of reaction times showed no significant session effect, *F* (1, 44) = 0.003, *p* = 0.958, *η* ^*2G*^ < 0.001, indicating no overall change in response speed. A significant condition effect was observed, *F* (2, 88) = 134.44, *p* < 0.001, *η* ^*2G*^ = 0.156, with significantly longer reaction times on incongruent trials compared to both congruent (*p* < 0.001) and neutral (*p* < 0.001) trials. The session × condition interaction was not significant, *F* (2, 88) = 1.38, *p* = 0.255, *η* ^*2G*^ < 0.001, suggesting that response time differences across conditions remained stable across sessions.

##### 3.3.2.2 Simon task (RT, AR), cog/incog/neut

Analysis of Simon task performance revealed no significant main effect of session (pre-vs-post) on accuracy, *F* (1, 44) = 1.44, *p* = 0.237, *η* ^*2G*^ = 0.016, indicating that overall accuracy did not significantly change following the intervention. However, a significant main effect of condition was observed, *F* (2, 88) = 34.50, *p* < 0.001, *η* ^*2G*^ = 0.108. *Post hoc* comparisons showed significantly lower accuracy on incongruent trials relative to both congruent (*p* < 0.001) and neutral (*p* < 0.001) trials. The session × condition interaction was not significant, *F* (2, 88) = 0.97, *p* = 0.381, *η* ^*2G*^ = 0.001, suggesting consistent performance patterns across sessions.

For reaction time, no significant main effect of session was found, *F* (1, 44) = 0.68, *p* = 0.412, *η* ^*2G*^ = 0.005. A significant condition effect emerged, *F* (1, 44) = 28.29, *p* < 0.001, *η* ^*2G*^ = 0.008, with faster responses for congruent trials compared to neutral trials. No significant session × condition interaction was observed, *F* (1, 44) = 1.02, *p* = 0.316, *η* ^*2G*^ < 0.001, indicating that response time differences between conditions remained stable from pre-to post-intervention.

##### 3.3.2.3 SDST (RT, AR)

**FIGURE 2:**
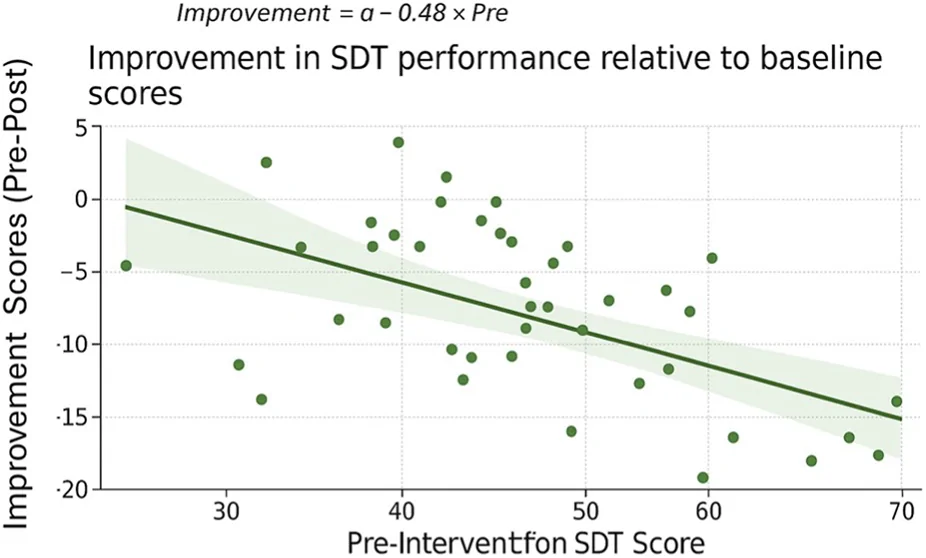
Baseline d scores predict magnitude of improvement following intervention. Improvement in Symbol Digit Substitution Test (SDST) performance as a function of baseline score. The negative correlation indicates that participants with lower pre-intervention SDST scores demonstrated greater post-intervention gains, suggesting that baseline cognitive performance moderated the magnitude of improvement. The shaded area represents the 95% confidence interval around the regression line.

No significant difference was observed between pre- and post-intervention performance on the SDST, *t* (44) = −0.13, *p* = 0.899, *d* = −0.02, indicating a negligible effect of the intervention on overall performance. However, a linear regression analysis revealed a significant positive relationship between pre- and post-intervention scores, *F* (1, 43) = 11.19, *p* = 0.002, *R* ^*2*^ = 0.207, suggesting that baseline scores were predictive of post-intervention outcomes. Further analysis of improvement scores (post minus pre) indicated a significant negative association with baseline performance, *F* (1, 43) = 19.24, *p* < 0.001, *R* ^*2*^ = 0.309. These findings suggest that participants with lower initial SDST scores experienced greater gains following the intervention (see Figure 2).

##### 3.3.2.4 Nonword repetition

Nonword repetition accuracy showed no significant pre–post change: scores decreased slightly from *M* = 9.64 (*SD =* 10.04) at T1 to *M* = 9.18 (*SD* = 9.24) at T2, *t* (44) = 0.78, *p* = 0.442, Cohen’s d = 0.12 (negligible effect). This pattern suggests minimal impact of the intervention on phonological repetition accuracy over the study interval.

##### 3.3.2.5 Phonemic fluency

**FIGURE 3:**
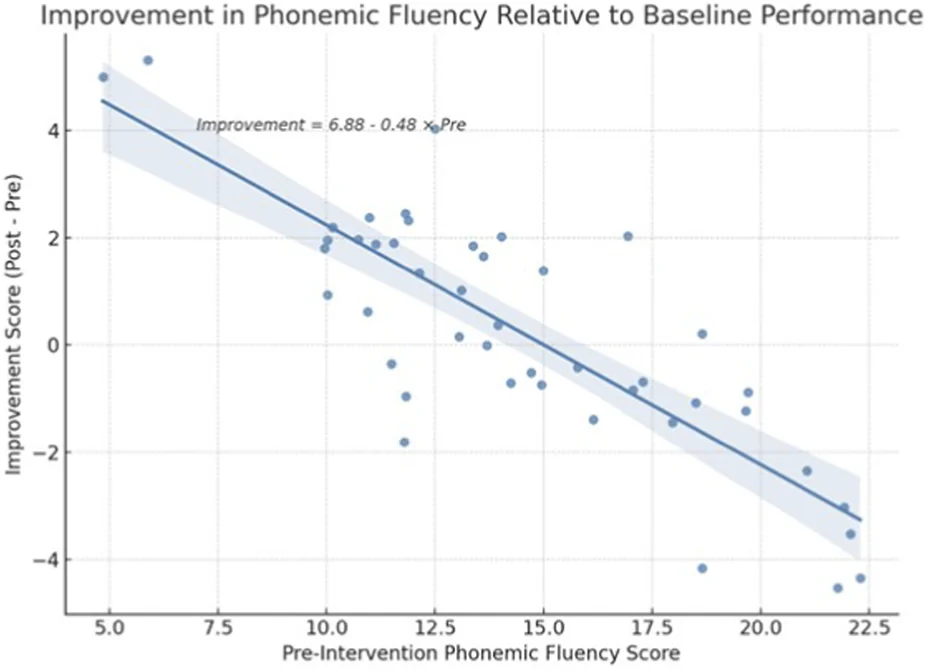
Improvement in phonemic fluency is inversely related to baseline performance. Improvement in phonemic fluency relative to baseline performance. A significant negative relationship indicates that participants with lower pre-intervention phonemic fluency scores showed greater post-intervention gains. The shaded area represents the 95% confidence interval around the regression line.

Phonemic fluency scores did not significantly differ between pre-intervention (M = 14.29, SD = 3.64) and post-intervention (M = 14.38, SD = 3.58), t (44) = −0.17, p = 0.865, d = −0.03, indicating a negligible group-level effect of the intervention on overall phonemic fluency performance. However, linear regression analysis revealed a significant positive association between pre- and post-intervention scores, *F* (1, 43) = 17.19, *p* < 0.001, *R* ^*2*^ = 0.286, with the regression equation: Post = 6.88 + 0.52 × Pre. This suggests that baseline performance was a strong predictor of post-intervention scores. Additionally, analysis of improvement scores (post minus pre) showed a significant negative relationship with baseline phonemic fluency, *F* (1, 43) = 14.09, *p* < 0.001, *R* ^*2*^ = 0.247, described by the regression equation: Improvement = 6.88–0.48 × Pre. These findings indicate that participants with lower initial phonemic fluency scores experienced greater improvements following the intervention (see Figure 3).

##### 3.3.2.6 Semantic fluency

**FIGURE 4:**
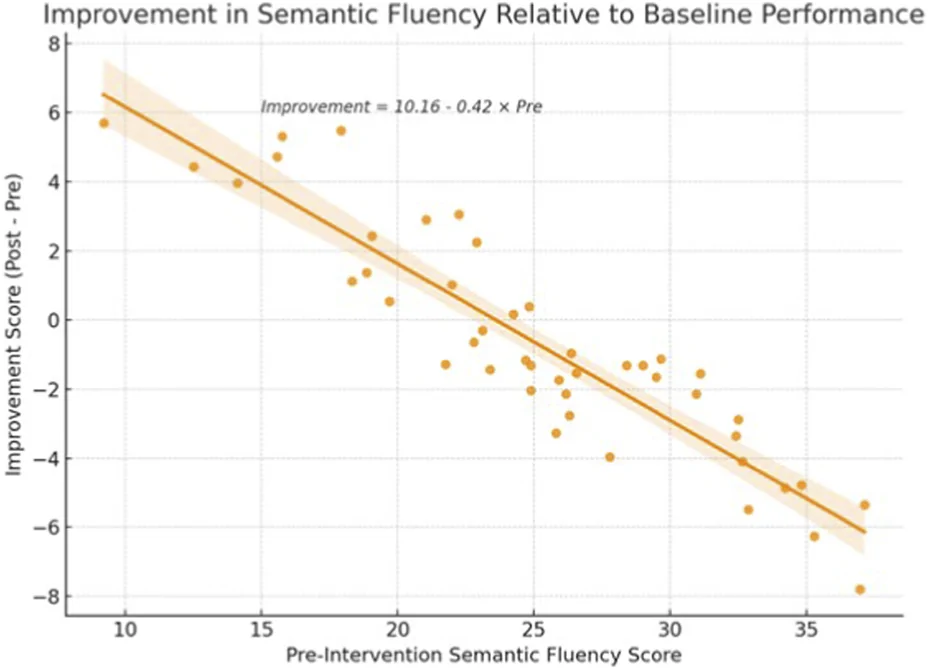
Pre- and post-intervention semantic fluency scores showing no overall mean change. A significant negative correlation indicates that participants with lower baseline semantic fluency scores exhibited greater post-intervention gains. The shaded region represents the 95% confidence interval around the regression line.

Semantic fluency scores did not significantly differ between pre-intervention (M = 23.49, SD = 5.41) and post-intervention (M = 23.80, SD = 5.31), t (44) = −0.43, p = 0.669, d = −0.06, indicating no reliable group-level effect of the intervention on overall semantic fluency. However, a linear regression analysis revealed a significant positive association between pre- and post-intervention scores, *F* (1, 43) = 23.17, *p* < 0.001, *R* ^*2*^ = 0.350, with the regression equation: Post = 10.16 + 0.58 × Pre. This suggests that baseline semantic fluency was a strong predictor of post-intervention performance. Additionally, analysis of improvement scores (post minus pre) demonstrated a significant negative relationship with baseline scores, *F* (1, 43) = 12.09, *p* = 0.001, *R* ^*2*^ = 0.219, with the regression equation: Improvement = 10.16–0.42 × Pre, indicating that participants with lower initial fluency scores exhibited greater relative improvement following the intervention (see Figure 4).

#### 3.3.3 Post hoc analyses

**FIGURE 5:**
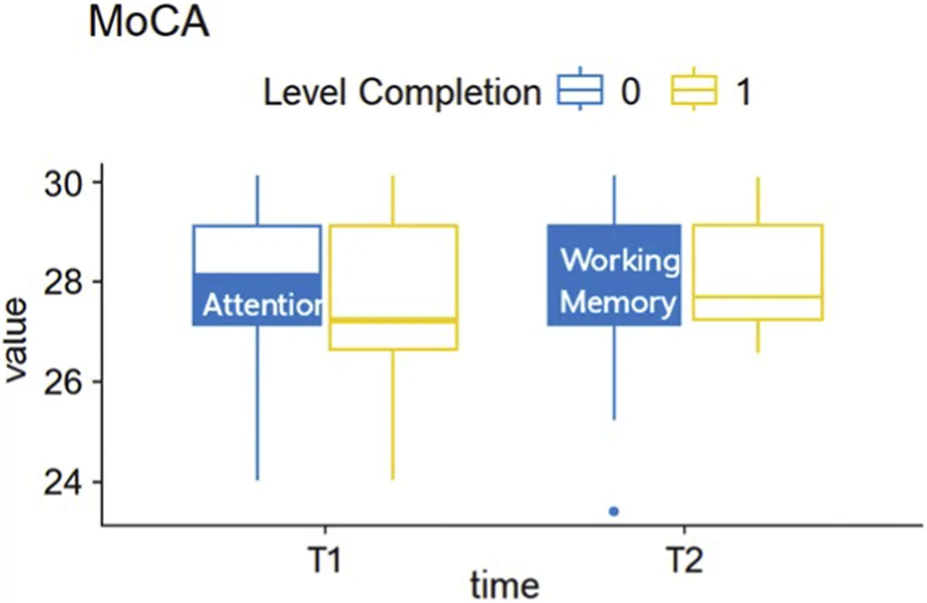
Effect of completing all training levels on cognitive improvement as measured by MoCA Boxplots show Montreal Cognitive Assessment (MoCA) scores at pre- (T1) and post-intervention (T2) for non-completers (0, blue) and completers (1, gold). Boxes indicate the interquartile range with medians as horizontal lines; whiskers span 1.5×IQR, and points denote outliers. Consistent with the regression results, completers exhibit higher MoCA scores at T2, reflecting greater post-intervention gains.

MMSE did not exhibit a significant pre–post difference in the subsample who completed all three levels of the L2 learning program. By contrast, MoCA change was 1.225 points higher in completers than in non-completers (Estimate = 1.225, *p* = 0.0025), consistent with an adherence-association pattern between program completion and cognitive gains. Within this subsample, age was not associated with change (Estimate = −0.038, *p* = 0.344), whereas male gender predicted smaller improvements (Estimate = −1.376, *p* = 0.0012), and the model explained 36.1% of variance in MoCA change (*R* ^*2*^ = 0.361). Additional *post hoc* tests examined domain-specific effects: semantic fluency gains increased with age (*β* = 0.3327, *p* = 0.0406); response times improved on the Stroop and SDST; and nonword repetition accuracy declined among completers (*β* = −0.089, *p* = 0.0377). Given the small sample and marked gender imbalance (≈one-third male), these *post hoc* analyses were designated as exploratory to test machine-learning results. See Figure 5 for the effect of completing all training levels on cognitive improvement as measured by MoCA.

#### 3.3.4 Machine-learning results

##### 3.3.4.1 Overall improvement patterns

**FIGURE 6:**
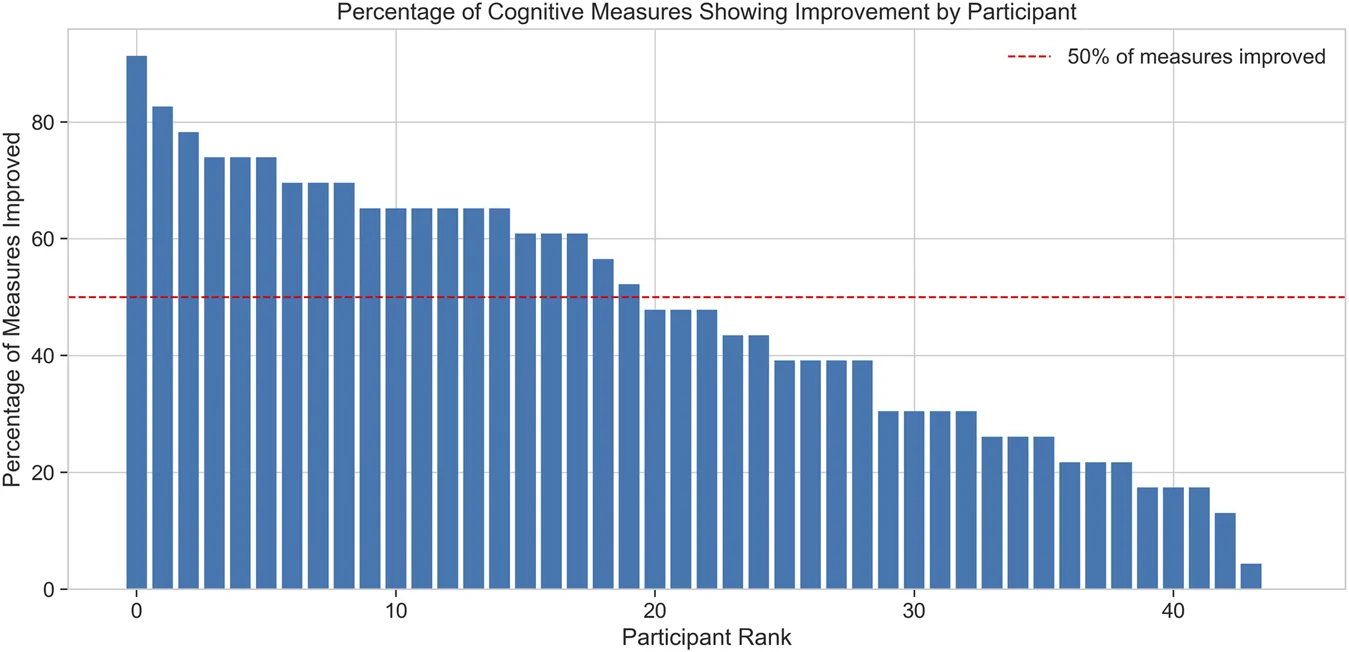
Percentage of cognitive measures showing improvement by participants. Each bar represents one participant, ranked from highest to lowest proportion of measures with positive pre–post change; the dashed red line marks the 50% benchmark. This visualization highlights heterogeneity in response, with some participants improving on most measures and others on relatively few.

The analysis of pre-to post-intervention changes revealed substantial variability in cognitive outcomes across participants (see Figure 6). Composite improvement scores ranged from −1.21 to 1.65 (*M* = 0.00, *SD* = 0.63), with 33.3% of participants exceeding the threshold for significant improvement (defined as a composite score >0.2 *SD*). The proportion of individual measures showing improvement per participant varied from 9.1% to 77.3% (*M* = 41.2%, *SD* = 17.8%). When examining performance by domain, reaction time (RT) measures in executive function tasks showed the most consistent gains. Notably, mean RT improvements were observed in the Stroop Incongruent (83.81 m, *SD* = 198.62), Stroop Congruent (37.67 m, *SD* = 178.45), and SDST RT (68.85 m, *SD* = 204.17) conditions. Accuracy rate (AR) improvements were smaller but detectable, with gains ranging from 0.012 (*SD* = 0.125) in Stroop Congruent AR to 0.074 (*SD* = 0.197) in Stroop Incongruent AR. We observed modest improvement for phonemic fluency (*M* = 1.08 words, *SD* = 3.64) and semantic fluency (*M* = 0.79 words, *SD* = 4.13). Measures of general cognitive status showed mixed patterns. Although some participants improved on the global screening measures, these gains were not uniform across the sample. MoCA showed only a very small mean increase (M = 0.09, SD = 1.44), whereas MMSE showed a mean decrease (M = −0.56, SD = 1.16). When examined at the individual level, 52%–67% improved on RT measures, 45%–59% on accuracy measures, 35.6% improved on MoCA, and 11.1% improved on MMSE. These patterns suggest that reaction time was the most sensitive marker of cognitive change, particularly in executive function tasks (see Figure 6).

##### 3.3.4.2 Predictors of cognitive improvement

**FIGURE 7:**
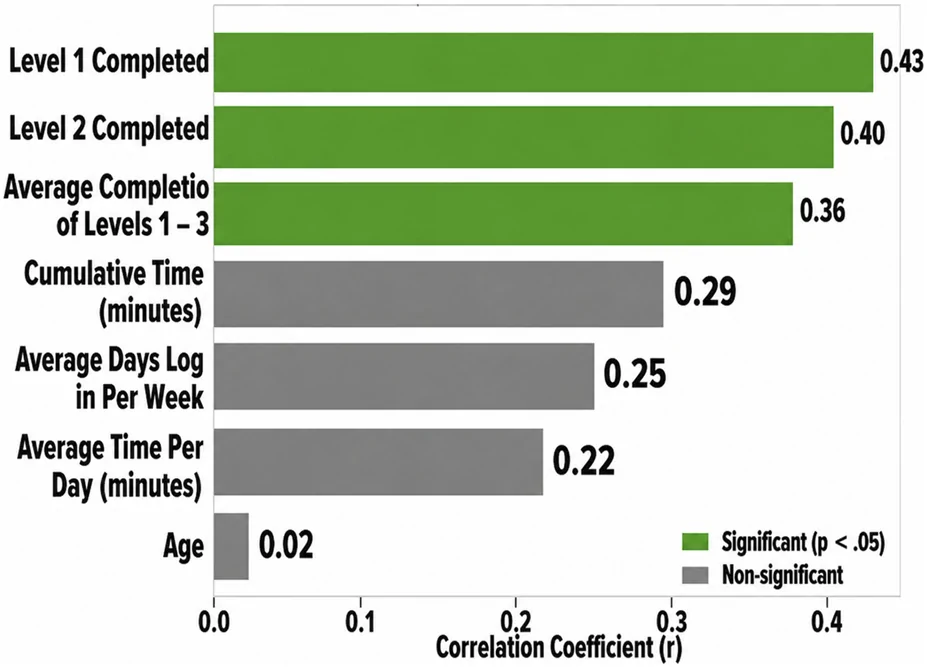
Top predictors of cognitive improvement. This horizontal bar chart ranks predictor variables based on their correlation with composite improvement. The x-axis represents the correlation coefficient (r), while the y-axis lists the predictor variables in descending order of strength. Green bars indicate statistically significant correlations (*p* < 0.05), while Gray bars represent non-significant relationships. The strongest predictors (program completion metrics) are highlighted at the top of the chart.

Correlational analyses revealed that program engagement metrics were meaningful predictors of cognitive improvement, with task completion emerging as the strongest factor. Specifically, completion of Level 1 was significantly correlated with composite cognitive improvement, *r* = 0.43, *p* = 0.003, 95% CI [0.17, 0.64], followed by Level 2 completion (*r* = 0.40, *p* = 0.007, 95% CI [0.13, 0.61]) and average completion across Levels 1–3 (*r* = 0.36, *p* = 0.014, 95% CI [0.08, 0.59]). Cumulative time spent on the program showed a trend-level association with improvement (*r* = 0.29, *p* = 0.054), while measures such as average days logged in per week (*r* = 0.25, *p* = 0.098) and Level 3 completion (*r* = 0.25, *p* = 0.100) were marginal. Other time-based metrics, including average weekly and daily time, were not significant predictors. Notably, average test completion was unrelated to cognitive improvement (*r* = 0.00, *p* = 0.986). These findings suggest that engagement quality, particularly in foundational levels, is a more robust predictor of improvement than raw usage time (see Figure 7).

Demographic analyses showed no evidence that age influenced cognitive improvement (*r* = 0.02, *p* = 0.883, 95% CI [−0.27, 0.31]). Gender differences were also non-significant: female participants (n = 32) had a mean improvement score of −0.04 (*SD* = 0.59), while males (n = 13) had a slightly higher mean score of 0.10 (*SD* = 0.70). An independent-samples t-test confirmed that this difference was not statistically significant, *t* (19.41) = −0.67, *p* = 0.512, d = 0.24, 95% CI [−0.61, 0.31]. The Bayes factor (*BF*_*10*_ = 0.38) provided moderate support for the null hypothesis, indicating that gender did not meaningfully affect intervention-related gains.

##### 3.3.4.3 Baseline performance and improvement

Analyses revealed a robust and consistent relationship between baseline performance and the magnitude of improvement across cognitive domains. For accuracy-based measures, lower initial performance was strongly associated with greater gains. Notably, the strongest negative correlations were observed for the Simon Neutral (*r* = −0.85, *p* < 0.001), Simon Congruent (*r* = −0.82, *p* < 0.001), and Stroop Neutral (*r* = −0.78, *p* < 0.001) accuracy rates. Similarly, strong inverse associations were observed for Nonword Repetition Accuracy (*r* = −0.73, *p* < 0.001), SDST Accuracy (*r* = −0.72, *p* < 0.001), and various averaged composite scores. Among global cognitive measures, Phonemic Fluency (*r* = −0.50, *p* < 0.001), MoCA (*r* = −0.48, *p* < 0.001), and Semantic Fluency (*r* = −0.47, *p* = 0.001) also demonstrated significant inverse correlations with improvement, while MMSE did not reach statistical significance (*r* = −0.27, *p* = 0.072).

Conversely, reaction time (RT) measures showed positive correlations with improvement, consistent with the definition of improvement as RT reduction. Strongest correlations were observed for Stroop Incongruent RT (*r* = 0.68, *p* < 0.001), average Stroop RT (*r* = 0.66, *p* < 0.001), and SDST RT (*r* = 0.56, *p* < 0.001). Similar trends were found across all Simon RT conditions (range: *r* = 0.54–0.55, all *p* < 0.001). These findings indicate that participants with slower initial reaction times tended to show the greatest RT reductions post-intervention.

**FIGURE 8:**
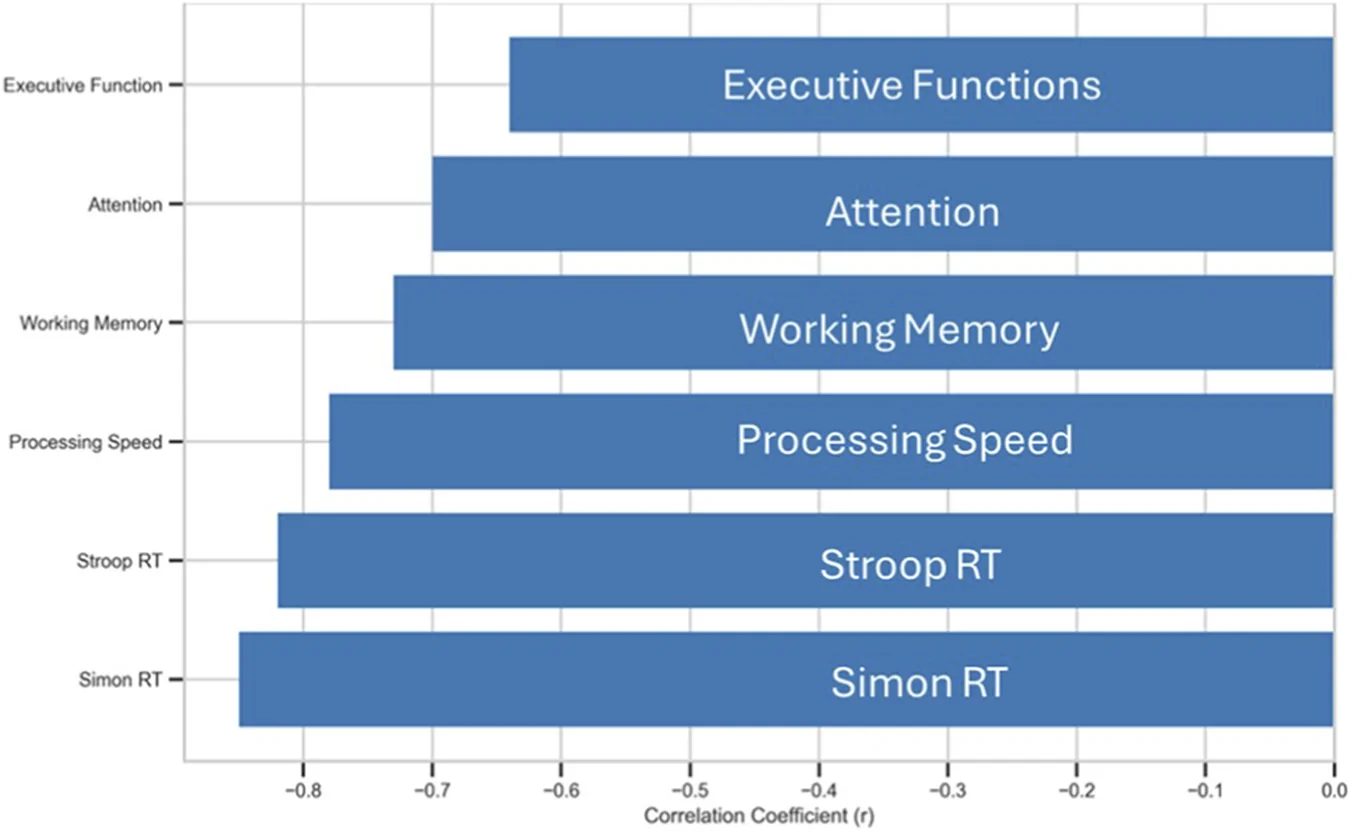
Baseline performance vs. improvement correlations this horizontal bar chart shows correlations between baseline performance and improvement for each cognitive measure. The x-axis represents the correlation coefficient (r), while the y-axis lists cognitive measures grouped by domain. Bars pointing left (negative values) for accuracy measures indicate that lower baseline performance predicts greater improvement.

In total, 13 out of 23 cognitive measures showed statistically significant correlations in the direction indicating that participants with lower baseline performance experienced greater improvement. The average absolute correlation coefficient across all measures was |r| = 0.62, reflecting a strong overall association between baseline status and intervention-related gains. See Figure 8 for baseline performance correlation with cognitive improvement.

##### 3.3.4.4 Cluster analysis

*K*-means clustering was conducted to identify distinct participant profiles based on cognitive improvement patterns. Silhouette analysis indicated that a two-cluster solution provided the best fit, yielding the highest silhouette score. The two-cluster model revealed clear differences in cognitive outcomes and engagement. Cluster 0 (*n* = 8; 17.8%) was characterized by large improvements in reaction time (*M* improvement = 329.93 m across RT measures) but minimal or negative changes in accuracy (*M* AR improvement = 0.008). Participants in Cluster 0 also had lower program completion rates, particularly for Level 1 (65.5% vs. 80.8% in Cluster 1), a lower mean age (65.38 years vs. 66.84), and a smaller proportion of male participants (12.5% vs. 32.4%).

In contrast, Cluster 1 (*n* = 37; 82.2%) exhibited moderate reaction time gains (*M* = 70.68 m) along with small but consistent improvements in accuracy (*M* AR = 0.13). This group also demonstrated higher engagement, including greater completion of program levels and more consistent use across all metrics. Principal component analysis (PCA) confirmed the distinctiveness of the two clusters: the first two principal components explained 42.7% of the variance, and the resulting plot revealed a clear separation between the clusters, supporting the presence of differential improvement trajectories within the sample.

##### 3.3.4.5 Profile of top improvers

An in-depth analysis of the top 25% of participants in terms of cognitive improvement (*n* = 11) revealed a distinctive demographic, engagement, and cognitive performance profile. The average age of top improvers was 66.18 years (*SD* = 4.51; range: 62–73), closely matching the full sample, with a gender distribution of 63.6% female and 36.4% male, slightly more balanced than the overall sample (71.1% female). In terms of engagement, top improvers demonstrated substantially higher program completion rates compared to other participants: Level 1 (*M* = 0.95 vs. 0.71), Level 2 (M = 0.87 vs. 0.63), and Level 3 (*M* = 0.60 vs. 0.43). They also logged in more frequently (M = 4.41 days/week vs. 3.85), spent more time on the program weekly (*M* = 310.57 min vs. 241.28), and accumulated greater overall usage (*M* = 5352.45 min vs. 4121.16).

Top improvers also had markedly lower baseline performance in executive function tasks, particularly on reaction time (RT) and accuracy rate (AR) measures. Compared to the rest of the sample, they exhibited RTs that were 140–174 m slower on Stroop Congruent, Stroop Incongruent, and Simon Congruent conditions. Accuracy was also lower by 8–24 percentage points in the same tasks. In contrast, top improvers showed comparable performance to others on verbal fluency (Phonemic: 14.82 vs. 14.12; Semantic: 23.09 vs. 23.62) and general cognition measures (MMSE: 29.55 vs. 29.71; MoCA: 27.55 vs. 27.79). These patterns suggest that greater gains were achieved by individuals who began with lower executive functioning performance but demonstrated high program engagement and task completion.

## 4 Discussion

We examined the cognitive effects of L2 learning in older adults living in the monolingual environment of Nebraska in a pre-post intervention study. Over 4 months, participants completed 60–90 min of training per day on at least 5 days per week using Rosetta Stone (Education Edition) in a language of their choice, enabling centralized monitoring of adherence and performance. Baseline demographics were collected, and both domain-specific and global cognition were assessed before and after the intervention.

As for global cognition, we did not observe pre-post improvement in MoCA or MMSE scores across the full cohort. Evidence regarding the cognitive effects of L2 learning in older adults remains mixed. For example, Ware et al. (2017) reported a modest, non-significant increase in MoCA scores following a four-month English-learning intervention in healthy older adults. In contrast, Bubbico et al. (2019) reported that MMSE scores changed modestly in the intervention group while declining in the control group following a four-month language-learning intervention, while Wong et al. (2019) observed improvements in overall cognitive performance, as measured by the Alzheimer’s Disease Assessment Scale–Cognitive Subscale (ADAS-Cog), following a six-month randomized controlled trial of L2 learning.

Several methodological differences may explain the variability in findings across studies. Among these, Bubbico et al. (2019) represents the most direct comparison to the present study, as both investigations examined four-month language-learning interventions and assessed global cognition using the MMSE. However, their study included a non-intervention control group, whereas the present study did not. In addition, participants in our cohort demonstrated higher baseline MMSE scores (MMSE = 29.67 ± 0.60) than those enrolled in the Bubbico intervention group (MMSE = 27.23 ± 1.72), potentially limiting the sensitivity of the MMSE to detect further improvement due to ceiling effects. Furthermore, Bubbico et al. implemented a teacher-led, classroom-based language program, whereas the present study used a self-directed Rosetta Stone program. More broadly, variation in participant characteristics, intervention format, training intensity, intervention duration, outcome measures, and analytical approaches likely contributes to the heterogeneous findings currently reported in the literature regarding later-life language learning and cognitive outcomes.

In addition, the highly educated nature of the sample may have contributed to elevated baseline cognitive reserve and strong baseline cognitive performance, therefore limiting the ability to detect measurable cognitive improvement over the intervention period. Higher education is commonly associated with greater cognitive reserve (Stern, 2009; Stern et al., 2020), which may have contributed to stronger baseline cognitive performance and reduced room for measurable improvement following the intervention. As a result, potential cognitive benefits of the intervention may have been attenuated by ceiling effects, particularly on global cognitive measures.

The full sample showed no statistical difference pre and post MoCA and showed a statistically significant decline in raw MMSE scores from pre-to post-intervention. While this finding indicates that the MMSE did not provide evidence of group-level cognitive improvement and complicates any broad claim regarding generalized cognitive benefit. However, the MMSE results require cautious interpretation as baseline MMSE scores were near ceiling in this cognitively healthy sample, with limited variability, reducing the instrument’s ability to detect improvement and increasing the influence of small item-level fluctuations. Regression analyses further suggested limited rank-order stability within this restricted range and a marginal pattern in which participants with lower baseline MMSE scores tended to show relatively more favorable change. Therefore, the MMSE decline may reflect ceiling effects, restricted range, measurement variability, and regression-related influences rather than a clear intervention-related decline. Nevertheless, this result underscores the need to avoid overgeneralizing the findings and supports the use of more sensitive cognitive measures and domain-specific executive-function tasks, in future controlled studies.

In our study, while we did not see homogenous results with global cognitive gain, we observed individual pre-post intervention improvements in MMSE and/or MoCA scores. In addition, interestingly, participants with lower baseline MMSE scores tended to show greater improvement. Consistent with the MMSE results, the MoCA showed a similar baseline-dependent pattern with larger positive intervention gains among participants with lower baseline performance. However, the overall pre–post change was short of strong statistical significance. Further, baseline cognition strongly predicted post-intervention performance, indicating that participants starting with a weaker cognitive status benefited most. These findings are noteworthy for two reasons. First, although most cognitively intact adults above the age of sixty remain stable within four to 6 months, and small practice effects are possible, some older adults show reliable MMSE or MoCA score change. Approximately 20%–27% of older adults with no diagnosis demonstrate measurable decline over 4–6 months, with at-risk or pre-MCI individuals often declining over even shorter intervals (Schönknecht et al., 2005). Second, these results point to the clinical effectiveness of L2 learning as a promising cognitive intervention, particularly for individuals who exhibit initial cognitive decline even over a relatively short interval (4 months). These observed changes are consistent with ceiling and floor effects (Yu et al., 2018; Jia et al., 2021; Kessels et al., 2022). Thus, high baseline performers showed attenuated gains due to limited headroom, whereas lower-performing participants exhibited larger improvements because they had greater room to improve. Proximity to ceiling constrains detectable gains; consequently, larger improvement scores are more likely among participants with lower baseline performance. These results are consistent with previous L2 learning studies in older adults reporting that only individuals with lower baseline cognition benefit more from L2 learning (Kliesch et al., 2021; Grossmann et al., 2023).

Among participants with lower baseline cognitive scores, improvement was greater on both measures, but only the MMSE showed a statistically significant positive association between baseline performance and subsequent change. This difference in responsiveness between the two instruments is not unusual; MMSE performance can be influenced by factors such as the ceiling effect in individuals with high cognitive functioning, as the test may not be challenging enough to detect progress in those already scoring near the maximum (e.g., MoCA ≥26–28, MMSE ≥28–30) (Trzepacz et al., 2015; Kessels et al., 2022). MMSE is also more susceptible to practice effects and general improvements (Calamia et al., 2012; Trzepacz et al., 2015). This is consistent with the MMSE’s coarser emphasis on orientation and basic attention, where early benefits of structured learning and test engagement can yield detectable changes (Folstein et al., 1975). By contrast, the MoCA targets subtler executive/visuospatial and memory processes (Nasreddine et al., 2005), which require greater training dose or time to shift total scores. Ceiling constraints in this relatively healthy sample further limited detectable change (Kessels et al., 2022; Ilardi et al., 2023). The MoCA demands more complex cognitive processing, and changes in total scores may require longer or more intensive interventions to become detectable (Cooley et al., 2015; Luo et al., 2022).

No significant pre-post intervention changes were found across specific cognitive domains, including the Stroop and Simon tasks, nonword repetition, phonemic and semantic fluency in group analyses. This suggests that domain-specific executive processes such as interference control and response inhibition may require a longer or more intensive intervention to elicit measurable improvement in cognitive control or processing speed. It may also reflect that the sample is too small to accommodate substantial individual heterogeneity in gains.

Specifically, regarding the Stroop task, although participants showed lower accuracy and slower reaction times in incongruent trials compared to neutral and congruent ones, a pattern known as the classic Stroop interference effect (Stroop, 1935), no significant pre–post changes were observed on the Stroop or Simon tasks. This suggests that interference-control processes may be less responsive to the present dose, intensity, or duration of the intervention or that these measures, administered online in a high-functioning sample, were insufficiently sensitive or underpowered to detect modest effects.

The absence of significant pre–post changes on the Simon task is consistent with findings from similar studies, which have also reported limited transfer effects of language learning interventions on interference control and other domain-specific executive functions as measured by the Simon task (Berggren et al., 2020; Grossmann et al., 2023; Meltzer et al., 2023; Schultz et al., 2024). However, several studies suggest that L2 learning can have a positive effect on cognitive performance in older adults as measured by the Stroop task (Pfenninger and Polz, 2018; Fong et al., 2022; Kliesch et al., 2022; Grossmann et al., 2023; Meltzer et al., 2023; Schultz et al., 2024). In our previous neuroimaging study conducted in 2024, improvements were observed in the Stroop task among monolingual older adults following a short language course in both accuracy and reaction time, as well as increased activation in brain regions associated with executive control (Schultz et al., 2024). Similarly, Bak et al. (2016) demonstrated that intensive language learning enhanced attentional networks and executive control, particularly among older participants. These findings also align with a randomized clinical trial in which older adults learned a new language using an app-based platform (Duolingo). They showed improvements on incongruent Stroop color-naming and 2-back accuracy (Meltzer et al., 2023). Finally, Grossmann et al. (2023) showed that only participants with lower baseline cognitive function experienced significant improvements in Stroop reaction time, suggesting that individuals with more room for improvement may benefit the most.

The modest effect observed for the Stroop but not the Simon task may reflect differences in task modality. The Stroop is language-based, and likely more sensitive to detecting inhibitory-control gains resulting from L2 learning. The Simon task, a nonverbal measure, may capture a different facet of executive control less influenced by linguistic processing. This pattern supports the view that cognitive control in bilingualism may be domain-specific, at least at low proficiency levels (Calabria et al., 2018), with language-related executive functions showing greater plasticity in response to linguistic experience than general, nonverbal control mechanisms.

Mean pre-post changes were not significant for the SDST, nonword repetition, and phonemic/semantic fluency. A previous study reported no significant pre–post changes after L2 learning in older adults in semantic and phonetic fluency (Bubbico et al., 2019). Previous studies did not use SDST or nonword repetitions, leaving room for future studies to explore if these tests are sensitive to pre-post intervention changes.

In our study, regression analyses revealed a baseline-dependent pattern where participants with lower initial scores showed greater relative improvement following the intervention. This suggests that verbal working-memory and lexical-retrieval processes are most responsive among those with greater capacity for improvement. This pattern is consistent with our findings on global cognition, reinforcing the observation that participants with lower baseline performance derived the greatest benefit from the intervention. This is also consistent with ceiling effects in higher-functioning participants, and that the intervention may be most beneficial for older adults with emerging cognitive decline, such as those with subjective memory complaints or MCI. Another important inference is that a longer or more intensive dose may be required to translate individual gains in cognitively healthy older adults into detectable group-level effects.

A primary observation was the heterogeneity of cognitive responses to the intervention. We applied machine-learning methods to answer three questions to clarify what drove change: 1) which participant characteristics and engagement metrics predicted improvement, 2) whether baseline performance modulated gains, and 3) whether distinct improvement profiles emerged. In this approach, we standardized all measure-specific gains (z-scores) and averaged them to form a composite index of global cognitive change. Participants were grouped into low/moderate/high tertiles based on the proportion of measures improved. This composite strategy, common in cognitive intervention research (Jaeggi et al., 2008; Shipstead et al., 2012; Melby-Lervåg and Hulme, 2013; Karbach and Verhaeghen, 2014; Moreau and Wiebels, 2021; van Dierendonck and Lam, 2023), reduced multiple testing, boosted power, and emphasized general (not task-specific) change. Results showed that while composite change scores across the sample ranged from clear gains to notable declines, about one-third of participants met the predefined threshold for meaningful improvement. Across domains, reaction-time indices in executive tasks (i.e., Stroop task and SDST) emerged as the most sensitive markers of change, with a majority (52%–67%) showing faster RTs, whereas accuracy, phonological and semantic verbal fluency, and global screens exhibited smaller, less frequent gains. Additionally, improvement on global screening measures was mixed, with 35.6% of participants improving on MoCA and 11.1% improving on MMSE. These findings suggest that in older adults, L2 learning may be more readily detected in processing-speed and executive-function measures than in broad global screening scores (Meltzer et al., 2023; Ware et al., 2017; Wong et al., 2019; Fong et al., 2022; Brouwer et al., 2025; Bubbico et al., 2025).

Correlational analyses demonstrated that engagement and learning progress, rather than total time spent, were the strongest predictors of cognitive improvement. The language learning program comprises three levels, with advancement contingent on achieving ≥85% on each lesson. Engagement as measured by lesson completion was significantly associated with greater cognitive gains, suggesting that strong engagement, especially with foundational content (level 1), drives the most benefit. These findings align with well-established literature demonstrating that systematic learning enhances cognitive performance, reinforcing the principle that structured practice yields measurable gains regardless of age and gender (McGaugh, 2000; Schneeweis et al., 2014; Srimaharaj et al., 2018). Indeed, demographic factors such as age and gender did not significantly influence outcomes, indicating that the intervention’s cognitive effects were consistent across participant subgroups regardless of age and gender.

Machine-learning regression analyses to predict improvement patterns based on engagement and time metrics revealed heterogeneous patterns of cognitive gain, driven by individual combinations of factors (see Figure 6). This may explain why any group effect appears masked, especially in small samples. Machine-learning results also confirmed our results yielded by the ANOVAs and regression analyses that intervention benefits concentrate among lower-performing individuals. Along the same line, across cognitive domains, results showed a strong inverse relationship between baseline status and subsequent gains, with the largest effects on accuracy and reaction-time indices in executive tasks (Stroop, Simon) and moderate effects for global cognition and verbal fluency. Results from the machine-learning approach confirmed our findings, indicating that baseline cognitive status is a key determinant of training-related improvement, with the greatest benefits emerging among those starting at lower performance levels. These results have been observed in previous studies conducted in a similar way (Kliesch et al., 2022; Grossmann et al., 2023). These results may suggest that older adults at the initial stages of cognitive decline benefit most from L2 learning intervention.

We applied K-means to change-score features to identify distinct participant profiles defined by their improvement trajectories and to further characterize heterogeneity in response. These clusters should be interpreted as preliminary response profiles rather than fixed clinical subtypes. The larger cluster was characterized by a more balanced pattern of cognitive change together with higher adherence and greater completion of language-learning levels, whereas the smaller cluster showed larger reaction-time gains with comparatively smaller accuracy improvements. This pattern suggests that response to second language learning may vary not only in magnitude but also in form, with some participants showing broader gains and others showing more selective speed-related change, potentially reflecting a speed-accuracy trade-off.

The K-means clustering results reveal meaningful variability in how participants responded to the L2 learning intervention. The emergence of two distinct clusters suggests that there were differential patterns of cognitive change rather than a uniform effect across the sample. Participants in the cluster representing the majority demonstrated moderate improvements in reaction time and accuracy rates on tasks measuring executive function, accompanied by higher engagement and program completion of language program levels. This suggests that consistent participation is important in balanced cognitive gains. In contrast, a smaller subgroup exhibited large reaction-time improvements but minimal accuracy gains, indicating potential speed-accuracy trade-offs or less stable performance gains, likely reflecting lower engagement and incomplete program exposure. The PCA confirmation of distinct clusters reinforces that individual differences in adherence and engagement play a critical role in shaping the nature and extent of cognitive benefits from L2 learning interventions.

Finally, an in-depth analysis of all variables identified a clear profile for the top quartile of improvers. These participants shared a clear profile: They all performed at a lower baseline executive-function performance (slower RTs and lower accuracy on Stroop/Simon). However, they all demonstrated substantially higher engagement as measured by completion of language learning across program levels. Demographically, they resembled the full sample, with only a slightly more balanced gender distribution with a higher percentage of women. Notably, their baseline verbal fluency and global cognition (MMSE, MoCA) were comparable to those of others, suggesting that executive deficits, not global status, primed the largest gains. First, these patterns confirm our findings that L2 learning benefits older adults at initial stages of executive dysfunction. These results are, in fact, in line with the literature on the cognitive advantage of lifelong bilingualism (Valian, 2015; Woumans et al., 2015; Degirmenci et al., 2022). In fact, there is evidence that lifelong bilingualism enhances executive functions at aging (Degirmenci et al., 2022). In a systematic review analyzing the existing literature, although the bilingual cognitive advantage seems to root from heterogeneous domains, subdomain analyses indicate a more reliable advantage in executive functions and inhibitory control. This advantage is particularly noticeable on Stroop and Simon tasks (Degirmenci et al., 2022). Secondly, it seems that to benefit from the intervention, it is important to have high adherence and engagement. This is in line with well-established literature on language and communication interventions. Treatment success is strongly linked to the frequency, intensity, and duration of practice (Wiseman-Hakes et al., 2018) and aphasia therapy (Husak et al., 2023; Brady et al., 2022), as well as more recent evidence on interventions using digital applications (Oakley-Girvan et al., 2022).

We conducted *post hoc* analyses to test whether high engagement is a determining factor in benefiting from the L2 learning intervention, and to support the machine-learning findings. Specifically, we examined the pre–post intervention changes in MoCA and MMSE as well as in specific domain measures, among participants who completed all three training levels. In this cohort, MMSE scores did not show a significant pre–post change; as expected, given that MMSE positive changes were more prominent only for participants with lower baseline scores. However, the expected change in MoCA was 1.225 points higher than in those who did not complete all levels (Estimate = 1.225, *p* = 0.0025), indicating an adherence-association pattern between program completion and cognitive gains. A 1.225-point increase on the MoCA represents a substantial and clinically meaningful change, given the test’s 30-point scale and its sensitivity to even small shifts in cognitive status. A 1.225-point difference in MoCA change should be interpreted cautiously but meaningfully within the context of a group-level intervention effect. Although this magnitude falls near the lower boundary of commonly cited SEM estimates for individual-level change on the MoCA; typically estimated at 1.2–1.8 points (Rossetti et al., 2011; Luis et al., 2009), those benchmarks were developed primarily for clinical interpretation of individual score reliability rather than for evaluating adjusted group-level differences in longitudinal regression models. Importantly, although the completion-associated MoCA difference remained statistically robust after Bonferroni correction, this finding should be interpreted cautiously because practice effects, self-selection, and unmeasured differences between completers and non-completers cannot be ruled out. Although practice effects remain a plausible limitation in the absence of an active control group, the pattern of findings is not readily explained by repeated test exposure alone. We argue that if MoCA gains primarily reflected practice effects, improvement would be expected to occur broadly across participants exposed to the same pre-post assessment procedures; instead, MoCA change was significant only among Completers. Moreover, the absence of comparable pre-post gains on other cognitive measures administered under the same testing conditions argues against a generalized retest-familiarity effect and suggests that the observed MoCA improvement may reflect training-related cognitive change rather than practice effects alone. This is also consistent with an adherence-association pattern between training exposure and cognitive change (Melby-Lervåg and Hulme, 2013). This pattern strengthens causal inference beyond simple pre–post improvement, suggesting that adherence and accumulated practice, not merely test familiarity (practice effect), contribute to measurable benefits in global cognition (Ware et al., 2017; Bubbico et al., 2019; Bubbico et al., 2025; Wong et al., 2019; Fong et al., 2022).

In this sub-sample, age was not associated with change; however, gender emerged as a significant predictor, with male participants showing smaller improvements, suggesting meaningful explanatory power of adherence and gender for cognitive outcomes. Although in line with the cluster analyses, this result should be interpreted cautiously, given the small sample size and the marked gender imbalance. Only one-third of participants were male, which limits statistical power and generalizability across sexes.

In addition, we observed age-related gains in semantic fluency, with older participants showing greater pre–post improvement, suggesting that lexical-semantic retrieval may be particularly amenable to enrichment in older adults (Llewellyn et al., 2009; Calatayud et al., 2023); perhaps via practice-related strategy use or increased engagement with language materials. Consistent with modest transfer to speeded processing and conflict resolution, response times improved on the Stroop and SDST, indicating faster attentional set and processing speed after training. In contrast, nonword repetition accuracy declined among completers, a pattern that could reflect fatigue or interference from increased focus on lexical learning over phonological rehearsal, measurement variability in the online setting, or a speed–accuracy trade-off.

In the nonword repetition, which measures phonological working memory (Baddeley, 1992; Baddeley et al., 1998; Gathercole, 2006), we observed a decrease in nonword repetition accuracy at T2 as compared to T1. Age and gender did not show any significant effects on this variation. This finding is particularly interesting and may be explained by two factors. One explanation is that as participants internalize the phonetic structure of a new language, their phonological system may begin to adapt, potentially interfering with performance on tasks relying on their native language’s phonology. This restructuring could temporarily impact their ability to process phonological information that does not conform to the patterns of the newly acquired language and recover as their L2 proficiency level increases as participants become more comfortable with the linguistic demands of the new language. Another possible explanation is that learning new grammar rules, as well as vocabulary, and articulating sounds in a new phonological system in a new language often demands significant mental effort (Ghazi Saidi et al., 2013; Ghazi-Saidi and Ansaldo, 2017a). Those who completed the full program may have experienced increased cognitive load, which could have led to a temporary decrease in phonological working memory capacity and accuracy. Nonword repetition tasks have been shown to be effective in detecting phonological memory deficits and language disorders, especially in pediatric populations. However, despite their diagnostic sensitivity, there is limited evidence supporting measurable improvements in nonword repetition following short-term interventions.

As for other domain-specific measures, in this cohort, similar to previous studies (Bubbico et al., 2019; Berggren et al., 2020), no significant changes were found in phonemic verbal fluency, response time, or accuracy on the Simon task, or response time on the Stroop task, either at the group level or across independent variables. This suggests that, within the current duration and intensity, the intervention did not produce measurable improvements in domain-specific executive or fluency processes. Participants with lower baseline scores on semantic and phonemic fluency showed more improvement post intervention, suggesting that individuals with lower cognitive performance gain more from L2 learning intervention. Phonemic fluency scores at baseline seem to be the best predictor for L2 learning intervention success.

The absence of significant changes in some measures may reflect several factors. Examples of such factors include ceiling effects on accuracy-based tasks, the limited sensitivity of broad screening instruments (e.g., MoCA or MMSE) to detect subtle short-term changes in healthy older adults, and substantial inter-individual variability that may obscure benefits at the group level. Additionally, some executive-function domains may require longer or more intensive training to produce reliable group-level effects. Further, the online versions of these tasks may have lacked sufficient sensitivity to detect subtle cognitive shifts in a high-functioning sample, even though the strong condition effects observed in the Stroop and Simon tasks support their construct validity.

An additional consideration is that most participants selected Spanish as the target language, with 39 of 45 participants choosing Spanish. This strong preference is notable and may reflect several factors, including perceived ease of learning, greater cultural familiarity, more frequent exposure to Spanish in the United States, or the relative linguistic proximity and orthographic transparency of Spanish for English-speaking learners compared with some other available target languages. Particularly, language distance plays a role on the cognitive demands of language learning (Ghazi Saidi et al., 2017; Ghazi Saidi et al., 2017a; Ghazi Saidi et al., 2017b) depending on the target language, including differences in phonology, orthography, grammar, vocabulary transparency, and perceived difficulty, the predominance of Spanish may have influenced participants’ engagement, rate of progress, and intervention outcomes.

It is also possible that the L2 learning intervention or L2 learning intervention using an online program such as Rosetta Stone, although cognitively engaging, was not sufficiently intensive, long, or targeted to produce measurable improvement on broad global cognitive screening measures. The intervention primarily engaged language learning, vocabulary acquisition, auditory-visual mapping, repetition, and communicative practice. Therefore, cognitive benefits may have been limited to domain-specific measures of lexical retrieval, phonological working memory, processing speed, attention, or executive control rather than in brief global screeners such as the MMSE or MoCA.

Any successful cognitive intervention depends on the duration, intensity, and frequency of training exposure (Klingberg, 2010). Sustained and consistent engagement allows for sufficient neural stimulation and consolidation of learning, while inadequate duration or irregular participation may limit measurable gains. In fact, greater exposure likely increases the frequency and duration of demands on attention, interference control, working memory, and lexical retrieval inherent to L2 learning, therefore intensifying experience-dependent plasticity (Zatorre et al., 2012). Clinically, we argue that these data demonstrate the importance of dose targets and adherence monitoring as core elements of language-based interventions for older adults. At the same time, alternative explanations warrant caution: completers may differ in unmeasured factors (e.g., motivation, baseline self-efficacy, time availability) that also predict improvement. Future trials should pre-specify intention-to-treat and per-protocol analyses, incorporate adherence-adjusted models, and test minimum effective doses to optimize feasibility and impact.

To summarize, global cognition (MoCA, MMSE) and the domain-specific measures (Stroop, Simon, nonword repetition, phonemic/semantic fluency) did not show significant group-level pre-post improvement. However, a *post hoc*, adherence-sensitive analysis indicated an adherence-associated pattern. Completers showed a +1.225-point advantage on MoCA over non-completers (*p* = 0.0025), a magnitude that is typically considered clinically meaningful on a 30-point scale and unlikely to reflect practice effects alone (Calamia et al., 2012; Cooley et al., 2015; Luo et al., 2022), reinforcing the role of training dose in cognitive change (Melby-Lervåg and Hulme, 2013; Kliesch et al., 2022).

We used machine-learning analyses to identify and weigh predictors of post-intervention change to clarify what drives improvement, providing a foundation for targeted dosing and personalization in future trials. Results from the machine-learning analysis suggest that L2 learning in older adults most strongly enhances processing speed, particularly in tasks engaging executive functions such as conflict management and selective attention (i.e., Stroop task and SDST); results that are consistent with previous similar work (Bak et al., 2016; Pfenninger and Polz, 2018; Grossmann et al., 2023; Meltzer et al., 2023; Schultz et al., 2024). In addition, the model suggested that global cognitive change was heterogeneous rather than uniform, with the clearer adherence-sensitive signal emerging for MoCA among completers rather than as a whole-sample effect across both screening measures. Thus, in line with previous literature, our results also indicated that global cognitive status in some participants may benefit from the intervention, reinforcing the broader potential of language learning as a means to support cognitive health in late adulthood (Ware et al., 2017; Ware et al., 2021; Bubbico et al., 2019; Wong et al., 2019; Fong et al., 2022).

Our findings reveal individually patterned cognitive gains shaped by diverse factor combinations. Such heterogeneity can obscure group-level effects, particularly in smaller samples. Machine learning Cluster analysis revealed that the top improver subgroup was characterized by lower baseline executive function (i.e., slower reaction times and reduced Stroop/Simon accuracy), paired with substantially higher engagement and completion of lessons across program levels. These gains were not attributable to global status: baseline verbal fluency and global cognition (MMSE, MoCA) were comparable to the rest of the sample, and demographics were similar, with a slightly higher proportion of women. The pattern indicates that in older adults, the largest cognitive benefits from L2 learning occur when adherence is high and there is room to improve executive control.

These preliminary findings suggest that participation in a structured, sustained L2-learning program may be linked to observable changes in cognitive performance over time among some older adults, particularly those with lower baseline scores and greater potential for measurable improvement. The results reflect that sustained demands on selective attention, interference control, and working memory during L2 learning practice plausibly engage frontoparietal control systems (Ghazi Saidi et al., 2013; Ghazi-Saidi and Ansaldo, 2017a), aligning with accounts of experience-based plasticity and cognitive reserve (Dash et al., 2017; Dash et al., 2021). Rather than indicating uniform gains on global screening across the whole cohort, the findings point to heterogeneous but potentially meaningful benefits that appear strongest under high-engagement conditions. Clinically, structured language learning programs emphasizing adherence, dose, and fidelity remain promising as accessible cognitive-enrichment tools for older adults, particularly for those at risk of cognitive decline.

## 5 Conclusion

In this study, we explored the cognitive effects of L2 learning in older adults (60–80 years), after 4 months of intensive intervention (5 days per week, 60–90 min per day). Whole-cohort effects on global cognition were mixed rather than uniformly positive. Global cognition (MoCA, MMSE) and the domain-specific measures (Stroop, Simon, nonword repetition, phonemic/semantic fluency) did not show significant group-level pre-post improvement. At the same time, participants who started at lower scores at baseline tended to show larger gains on several measures, indicating that baseline cognitive status shaped response to the intervention.

Overall, the results suggest that even a four-month L2 learning intervention may yield measurable but selective cognitive benefits in some older adults. Rather than a uniform group-level improvement across all measures, different patterns were observed in different measures that may suggest that the L2 learning cognitive effects are domain-specific and/or that different screening tools vary in sensitivity to change. Sustained L2 learning may engage selective attention, interference control, and working memory, supporting frontoparietal control systems and aligning with experience-based plasticity and cognitive reserve frameworks. Further, change was concentrated in participants with greater room to improve at baseline and in those who maintained higher adherence to the program. The clearest adherence-sensitive signal was the +1.225-point MoCA advantage among completers relative to non-completers, whereas group-level effects on several other measures remained limited. These findings support the potential of language learning as an accessible and engaging form of cognitive enrichment in older adults, especially those with lower cognitive performance. However, the results indicate that benefits may be adherence or dose dependent, modest at early time points, and distributed unevenly across cognitive domains, with screening tools and executive measures differing in sensitivity to short-term change. Clinically, our results support that structured language learning programs that emphasize adherence, dose, and fidelity remain promising as accessible cognitive-enrichment interventions for older adults, especially for those with emerging decline or lower baseline performance.

## 6 Limitations and future directions

Although the study yielded promising outcomes, important limitations must be acknowledged when interpreting the results. The sample was highly educated, with all participants having completed at least a college education. This limits the generalizability of the findings to older adults with lower levels of educational attainment. Self-selection and adherence bias are likely: participants who completed all levels may differ on unmeasured factors such as motivation, baseline self-efficacy, health status, time availability, personality type, or digital fluency; factors that also promote improvement. Measurement constraints (ceiling effects on global screens, practice effects, and limited domain granularity) may inflate or obscure change. The single-site, monolingual sample and modest size limit generalizability, and heterogeneous language choices/doses introduce treatment non-uniformity. Without randomization, blinding, or active control, we cannot exclude Hawthorne effects, regression to the mean, or the effects of test repetition. The absence of a control condition limits our ability to definitively attribute the observed pre-post changes to the language learning intervention itself. Without a comparison group, alternative explanations such as practice effects associated with repeated cognitive testing, increased engagement in cognitively stimulating activities during the intervention period, or other nonspecific factors cannot be fully excluded. Future research should therefore incorporate well-matched active control conditions to more clearly isolate the specific contribution of second language learning to cognitive outcomes and to establish causal inferences regarding its potential role as a cognitive intervention in aging. While gender-related analyses were performed and discussed, these results should be interpreted within the context of exploratory analyses rather than definitive conclusions, as the present study was not adequately powered to examine subgroup differences. Larger-scale studies will be necessary to evaluate potential gender effects more reliably and more diverse educational backgrounds to determine whether L2 learning may have different cognitive effects among older adults with lower baseline cognitive reserve or greater vulnerability to cognitive decline. Missing data and analyses limited to completers may introduce attrition bias, and the exploratory machine-learning analyses would benefit from confirmation in larger independent samples to establish the stability and generalizability of the response profiles identified here.

To address these issues, future trials should pre-specify intention-to-treat and per-protocol analyses, incorporate randomization with active, dose-matched controls (e.g., matched digital training), and standardize dose (duration, intensity, frequency). Future studies should consider using a more sensitive global cognitive composite, such as the NIH Toolbox Cognition Battery Total Cognition Composite or the RBANS Total Scale Index, while retaining domain-specific measures of executive control, processing speed, working memory, and language. Outcomes should be chosen to minimize ceiling/practice effects, include domain composites and multimodal measures (executive tasks, everyday function, and, where feasible, neuroimaging), and studies should be powered for subgroup/interaction tests. Future studies should incorporate longitudinal follow-up assessments to determine whether cognitive changes associated with second language learning are sustained over time and whether such interventions may contribute to longer-term mitigation of age-related cognitive decline. Moreover, given our strictly monolingual context and the online delivery of language learning, replication in more diverse settings will be essential to evaluate reach, equity, and generalizability. Finally, future studies should include a more balanced distribution of target languages or directly examine target linguistic distance as potential moderators of cognitive and language-learning outcomes.

## Data Availability

The datasets presented in this article are not readily available because the project has not concluded. Data will be made available once the project concludes and all manuscripts in-preparation based on this dataset are published. Requests to access the datasets should be directed to ghazisaidil2@unk.edu.
